# Translation, Cross-Cultural Adaptation, and Validation of the Malay-Version of the Factors Influencing Community Willingness to Perform Cardiopulmonary Resuscitation and Use an Automated External Defibrillator Questionnaire

**DOI:** 10.3390/ijerph19084882

**Published:** 2022-04-17

**Authors:** Amsyar Daud, Azmawati Mohammed Nawi, Azimatun Noor Aizuddin, Mohammad Fadhly Yahya

**Affiliations:** 1 Department of Community Health, Faculty of Medicine, Universiti Kebangsaan Malaysia, Kuala Lumpur 56000, Malaysia; amsyardaud@yahoo.com.my (A.D.); azimatunnoor@ppukm.ukm.edu.my (A.N.A.); 2 Emergency and Trauma Department, Hospital Melaka, Jalan Mufti Haji Khalil, Melaka 75450, Malaysia; fadhlyyahya@moh.gov.my

**Keywords:** translation, validation, cardiopulmonary resuscitation, automated external defibrillator, Malay

## Abstract

Limited factors influence community willingness to perform cardiopulmonary resuscitation and use an automated external defibrillator, making it difficult to take preventive and control measures to improve the survival of patients with out-of-hospital cardiac arrest. In this study, we translated and evaluated the Malay-language version of the cardiopulmonary resuscitation and an automated external defibrillator questionnaire. The translation and evaluation involved three phases: development, translation and cultural adaptation, and validation. Content validity was assessed by five experts, and demonstrated a content validity index of 0.98 and a Fleiss kappa index of 0.159. Construct validity for the multi-item scale performed using factor analysis and involving 100 participants was 0.777. Factor analysis using the varimax rotation method demonstrated the appropriateness of the data in the exploratory factor analysis. Cronbach’s alpha was 0.849, suggesting high reliability. Test–retest reliability involving 45 participants calculated using the intraclass correlation coefficient had a value of 0.723. The findings demonstrate that the Malay-version FIXED questionnaire is a valid and reliable instrument and is ready to be used by health care workers and policymakers to evaluate the factors influencing the community’s willingness to perform cardiopulmonary resuscitation and use an automated external defibrillator.

## 1. Introduction

Out-of-hospital cardiac arrest (OHCA) is a major health problem globally in terms of morbidity and mortality, with low survival rates related to community health [1,2]. Out-of-hospital cardiac arrest is defined as a condition in which cardiac mechanical activity suddenly ceases affecting hemodynamic conditions, as evidenced by the absence of signs of life outside the hospital [3,4]. The incidence is highly time-dependent and its high mortality is associated with a wide time gap between cardiac arrest and emergency medical services (EMSs) arrival [5]. Cases involving OHCA are the leading cause of death in the world, with over 135 million deaths recorded annually, especially in developing countries [1,6]. Globally, incidences involving OHCA involve 20–140 per 100,000 people, and the survival rate of patients with OHCA is only 2–11% [2,7]. Therefore, reducing the burden of OHCA-related diseases is a critical community health problem and should be addressed with constructive measures. The survival of OHCA patients during those critical minutes before EMS team arrival depends on the willingness of the bystanders close to the victim to perform cardiopulmonary resuscitation (CPR) until EMS assistance takes over [8]. CPR performed by the community can help improve OHCA patient survival, provide better quality of life, and lead to higher hospital discharge rates [9,10,11].

The factors influencing community willingness to perform CPR and use an automated external defibrillator (AED) prior to EMS team arrival are limited, rendering it difficult for preventive and control measures to be taken to improve the survival of OHCA patients. To date, no standard instrument has been developed to assess the factors influencing the community’s willingness to perform CPR and use an AED in an emergency situation. The purposes of this study were to translate and evaluate the Malay-language version of the Factors Influencing Community Willingness to Perform CPR and Use an AED (FIXED) questionnaire. The FIXED is the author’s invention to explore the factors that influence community willingness to perform CPR and use an AED and has never been described before. There is a need to translate the questionnaire into Malay, the official language of Malaysia. As a result of the translation, cultural adaptation, and validation, we will be able to examine previously identified factors such as knowledge, training, perceptions, attitudes, perceived norms, self-efficacy, intentions, behaviours, and their barriers [5,12,13]. We report the translation and cultural adaptation, validation using content validation (content validity index, CVI; Fleiss kappa index, FKI), construct validation (exploratory factor analysis, EFA; principal component factor analysis, PCA), and reliability using internal consistency (Cronbach’s alpha) and test–retest reliability (intraclass correlation coefficient, ICC).

## 2. Methods

**Figure 1:**
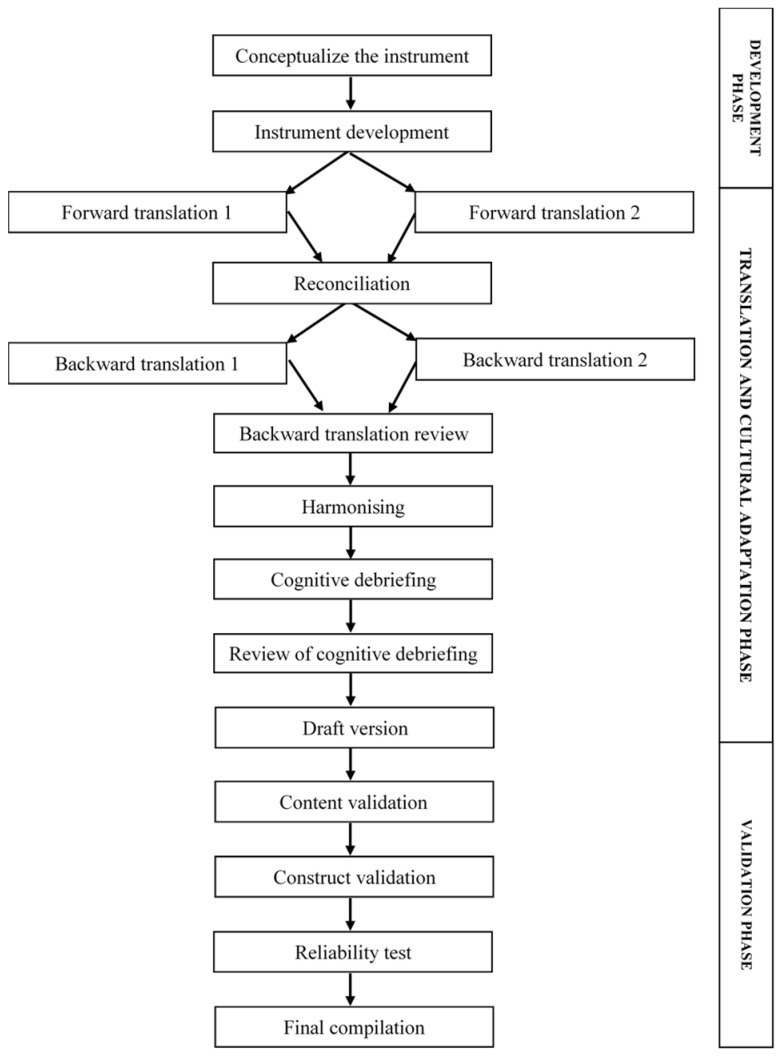
The cross-cultural adaptation and evaluation process.

The cross-cultural adaptation and evaluation process was designed by adapting the instrument development process proposed by Mohamad Marzuki et al. [14] and Wild et al. [15] (Figure 1). The process involves 14 steps grouped into three phases: development, translation and cultural adaptation, and validation.

### 2.1. Phase 1: Development Phase

**Figure 2:**
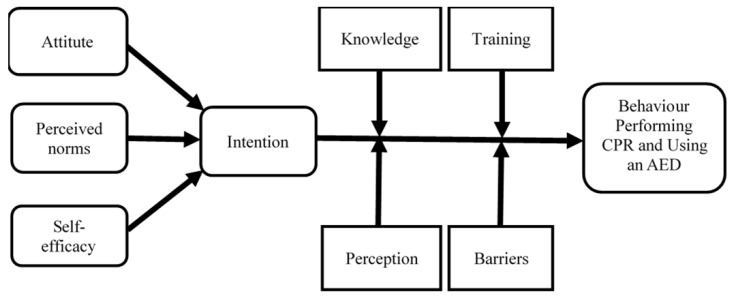
A conceptual framework of the community willingness to perform CPR and use an AED.

The items were generated based on a comprehensive literature review to identify publications assessing factors influencing the community’s willingness to perform CPR and/or use an AED. The first step is identifying the constructs or domains to be measured. Constructs were selected using the ‘intention-focused model for bystander CPR performance’ [16] and the ‘theory of planned behaviour with background factors (TPB)’ [17]. Based on the two theoretical frameworks and coupled with the literature review, nine constructs of instruments for measuring the factors influencing community willingness to perform CPR and use an AED were identified: (1) knowledge, (2) training, (3) perception, (4) attitude, (5) perceived norms, (6) self-efficacy, (7) intention, (8) behaviour, and (9) barriers. A conceptual framework (Figure 2) was constructed based on both theories to illustrate the relationship between the constructs of the variables to be studied.

The FIXED questionnaire was developed based on the above nine constructs using the deductive and inductive methods. The deductive method was used by adapting from the literature (i.e., knowledge of CPR and AED) [18,19] the attitudes, perceived norms, and self-efficacy [12]; intention [19]; and perceptions, barriers, and behaviours [20]. The inductive method was conducted through meeting with two community health experts to discuss the questionnaire contents, determine the appropriateness of the items to be used, and decide whether to include items to be used. The final decision was made based on the consensus reached with the researcher.

### 2.2. Phase 2: Translation and Cultural Adaptation Phase

In this phase, two bilingual translators with the skills to translate the questionnaire from the original English into the Malay with general terms that are easily understood in Malaysia performed the forward translation. The conciliation stage involved determining the appropriate forward translation and changing items in the forward translation or changing the forward translation completely if necessary, reconciling and harmonising the Malay version of the instrument. Any discrepancies were resolved with intensive discussions among the experts and translators. A written report recording all synthesis processes, with each issue addressed and how they were resolved, was produced (Supplementary Materials Table S1).

The backward translation involved translating the reconciled questionnaire (Malay) back to the original language (English). To review the backward translation, the backward translated (English) items were compared with the original English version to identify any equivalent conceptual differences. Both the English and Malay versions were refined to reach agreement on a satisfactory equivalent version of the instrument.

In the harmonising stage, all translation items in the desired language were compared with the source version. Any discrepancies were resolved with intensive discussions among the experts and translators. At the cognitive briefing stage, pre-draft version of the questionnaire was field-tested among the target population. Ten respondents were recruited to answer the pre-test questions of the Malay version FIXED questionnaire, which was distributed online. The respondents, who were randomly selected based on their participation in the Melaka CPR Fun Run programme between 2018 and 2020, were at least 18 years old, Malaysian citizens, understood Malay, and were residents or permanent residents of Melaka State.

The cognitive debriefing review was guided by the respondents’ feedback. The researcher and supervisor agreed via consensus to make changes to the items in terms of word substitution, word deletion, word addition, and sentence structure. The Malay translation was modified carefully to maintain the conceptual meaning of the item and the translation was finalised through discussion with the researcher and supervisor. The draft version would undergo validation before the FIXED instrument was finalised.

### 2.3. Phase 3: Validation Phase

#### 2.3.1. Content Validity

These expert ratings were calculated using content validity index (CVI) to measure consensus, the content validity of individual items (I-CVI) to measure proportional agreement, and Fleiss Kappa (Cohen kappa adaptation for >3 evaluators) to measure expert agreements [21,22,23]. In the present study, the expert review panel comprised five people with various background specialisations, namely, cardiology, emergency medicine, curriculum development, CPR and AED instruction, and patient education (Supplementary Materials Table S2). The experts were required to evaluate the instrument, critically review the constructs and items, and assess the overall presentation of the instrument before scoring each item. Once they had finished reviewing the constructs and items, the experts were required to assign scores to each item independently based on the relevant evaluation scales.

Four levels of suitability for the questions and the answers were assessed on a 4-point scale: 1 = the item is not relevant to the measured construct; 2 = the item is somewhat relevant to the measured construct; 3 = the item is quite relevant to the measured construct; and 4 = the item is highly relevant to the measured construct [24]. Prior to CVI calculation, the relevance rating was recoded as 1 (rating scale, 3 or 4) or 0 (rating scale, 1 or 2). The CVIs were calculated at the item level (I-CVI) and scale level (S-CVI). The I-CVI was calculated as the number of experts who assigned a score of 3 or 4 divided by the total number of experts (22). Two indices were calculated to derive the S-CVI: (1) the proportion of items on a scale that the expert scores as valid (rating 3 or 4) (universal agreement (UA) by experts = S-CVI/UA), and (2) the average share of items on a scale rated 3 or 4 (average agreement (Ave) by experts = S-CVI/Ave) [25]. An I-CVI of >79% indicates that the item is appropriate, between 70% and 79% indicates that the item requires revision, and I-CVI of <70% indicates that the item should be eliminated [26].

After the expert panel had reviewed the items, their responses were analysed using the FKI due to its suitability to evaluate a large number of raters [27]. Based on the interpretation of the Fleiss kappa [28], κ ≤ 0 indicates that there is no agreement among the raters, κ between 0.0 and 0.20 indicates slight agreement, κ between 0.21 and 0.40 indicates fair agreement, κ between 0.41 and 0.60 indicates moderate agreement, κ between 0.61 and 0.80 indicates substantial agreement, and κ between 0.81 and 1.0 indicates almost perfect agreement.

#### 2.3.2. Construct Validity

EFA was performed to determine how items on the FIXED questionnaire correlated with each other and whether they could be grouped into sub-constructs. A hundred respondents were recruited to answer the Malay version of the FIXED questionnaire. The respondents, who were randomly selected based on their participation in the Melaka CPR Fun Run programme between 2018 and 2020, were at least 18 years old, Malaysian citizens, understood Malay, and were residents or permanent residents of Melaka State. The sample size for EFA was calculated using an online Cronbach’s alpha hypothesis testing calculator, which is available online at https://ptenklooster.nl/psychometric-sample-size-calculators/cronbachs-alpha-hypothesis-testing/ (accessed on 6 April 2022) [29,30]. Based on an expected Cronbach’s alpha of 0.80 for 59 items in a FIXED questionnaire and a desired power of 0.8 (80%), 100 subjects were needed to demonstrate that this Cronbach’s alpha value was significantly different from a minimum acceptable Cronbach’s alpha value of 0.70 at a significance level of 0.05 (two-tailed significance).

The sampling adequacy was measured between 0 and 1 with Kaiser–Meyer–Olkin (KMO) assessment, unbiased estimates of actual factor scores were produced using Bartlett’s test of sphericity, the convergent validity of the items was demonstrated using communalities, and the number of factors to be retained was determined using the scree plot test [31,32,33]. A preliminary KMO test performed to determine the adequacy of sampling suggested 0.6 as the minimum value for factor analysis and interpreting KMO values; a value between 0.60 and 0.70 was considered adequate, that between 0.7 and 0.8 was good, that between 0.8 and 0.9 was great and a value of >0.9 was considered superb [33]. Item communalities were considered unrelated to each other if they had a value of <0.40, while a value of 0.40–0.70 indicated low to moderate communality and a value of >0.8 indicated high communality [34].

After confirming the suitability for factor analysis, factor extraction was performed using PCA. The factorability of the 44 multi-item scale was assessed to determine the suitability of the data for use in the EFA using the following tests: eigenvalue rule >1, at least 50% cumulative percentage of variance (CPV) extracted, and the scree plot test [35]. EFA was performed again using the rotation method to simplify and clarify the data structure through varimax orthogonal rotation to produce unrelated factors [36]. Varimax rotation factor matrix analysis with factor loading with a significant loading value set at 0.32 was used as the criterion to determine whether an item would be removed, and each component was evaluated to ensure that it had at least three items with loading >0.4 [37,38].

Reliability can be assessed using internal consistency, test–retest reliability, and inter-rater reliability [39]. Internal consistency is estimated using Cronbach’s alpha coefficient [40]. Cronbach’s alpha of 0.70 has been proposed to indicate sufficient internal consistency and for exploratory or pilot studies, it is suggested that the Cronbach’s alpha reliability should be ≥0.60 [41,42]. Cut-off points for corrected item–total correlation >0.30 are acceptable [43]. The four benchmarks used for reliability are: excellent reliability (>0.90), high reliability (0.70–0.90), moderate reliability (0.50–0.70), and low reliability (<0.50) [44].

The test–retest reliability was assessed by calculating the ICC. ICC values <0.5 indicate poor reliability, that between 0.5 and 0.75 indicate moderate reliability, that between 0.75 and 0.9 indicate good reliability, and that >0.90 indicate excellent reliability [45].

## 3. Results

### 3.1. Sociodemographic Characteristics of the Participants

The mean age of participants was 31.43 years (SD = 9.01, range: 18–63). The gender ratio was almost 1:1 and most participants had a higher education background (85%), were married (53%), lived in city residential areas (61%), and were from the B40 group (household income <RM 4850) (59%). Most participants did not have any medical history of chronic illness (97%), no medical history of family members with chronic illnesses (66%), no experience of witnessing the incidence of cardiac arrest (62%), and had never performed CPR on a victim of cardiac arrest (65%) (Table 1).

### 3.2. Conceptualisation of FIXED

Based on deductive and inductive methods, 59 items in nine constructs were generated and prepared for the validation process. The conceptual and operational definition of the instrument was defined for each construct (Supplementary Materials Table S3). All items were previously written in Malay as it was easier for all experts and respondents to understand.

### 3.3. Content Validity

All content validity (CVI) calculations were from the FIXED questionnaire development (nine constructs, 59 items). Five experts performed the relevant assessments on the item scale (Supplementary Materials Table S4). No item dropped had an I-CVI value < 0.79. The average I-CVI rated as relevant across the five experts was 0.98. Table 2 shows the five experts’ construct-based CVI. The averaging calculation method (S-CVI/Ave) obtained for all constructs was 0.98, while the universal agreement calculation method (S-CVI/UA) was 0.90. The S-CVI/UA was 1.00, 1.0, 1.0, 0.38, 0.75, 1.0, 1.0, 1.00, and 1.0 for constructs 1, 2, 3, 4, 5, 6, 7, 8, and 9, respectively. Based on the calculations, it can be concluded that the I-CVI, S-CVI/Ave, and S-CVI/UA were satisfactory. The Fleiss kappa statistic had a kappa value of 0.159 (95%CI: 0.047, 0.268, *p* = 0.001) (Supplementary Materials Table S5), which indicates little agreement [28].

### 3.4. Construct Validity

The KMO value of 0.777 obtained from the data indicated good correlation between items and factor analysis was appropriate for this dataset. The *p*-value for the Bartlett test of sphericity was significant (χ^2^ (946) = 3346, *p* < 0.001), indicating that there was appropriate correlation between items based on the correlation matrix. After the overall suitability of the items for factor analysis had been confirmed, construct-based analysis was performed. A KMO value of 0.802 was obtained for perception, 0.643 for attitude, 0.582 for perceived norms, 0.786 for self-efficacy, 0.854 for intention, 0.818 for behaviour, and 0.812 for the barriers. All constructs had KMO values >0.6, except for the perceived norms (KMO = 0.582). The Bartlett test of sphericity clearly showed that all constructs had significant values (*p* < 0.001) (Table 3).

The communality values for all 44 items on the multi-item scale were 0.296–0.853 (Supplementary Materials Table S6). However, one item had a communality value of <0.4: “knowing the importance of starting a resuscitation before EMS arrival” (0.296), which was considered unrelated to other items [36]. The other 43 items had communality values between 0.454 and 0.853, indicating that the relationship with other items had low to moderate communality.

EFA was performed again using the varimax orthogonal rotation method for the seven constructs (i.e., perception, attitude, perceived norms, self-efficacy, intention, behaviour, and barriers) (Table 4). All items in all constructs had a factor loading >0.4 and were considered acceptable [46]. The factor loading for the perception construct revealed three factors with an eigenvalue of >1 and 72.8% CPV. The first, second, and third component had a variance value of 41.2%, 18.8%, and 12.7%, respectively. Two items (9 and 10) were loaded on one component. The extracted perception sub-constructs were labelled Factor 1, “AED implementation strategies”; Factor 2, “community perception on the importance of CPR and AED”; and Factor 3, “community perception on AED handling training”.

For the attitude constructs, three factors with an eigenvalue of >1 with 69% CPV were extracted for the FIXED attitude sub-construct. The first, second, and third component had a variance value of 31.2%, 24.4%, and 13.3%, respectively. One item (12) was loaded on one component. The extracted attitude sub-constructs were labelled Factor 1, “fearful of CPR and AED”; Factor 2, “courage for CPR and AED”; and Factor 3, “importance of CPR and AED”. For the perceived norms construct, two factors with an eigenvalue of >1 and 70.9% CPV were extracted. The first and second component had a variance value of 44.6% and 26.3%, respectively. One item (22) was loaded on one component. The sub-constructs of the perceived norms extracted were labelled Factor 1, “relatives” and Factor 2, “community”.

**Figure 3:**
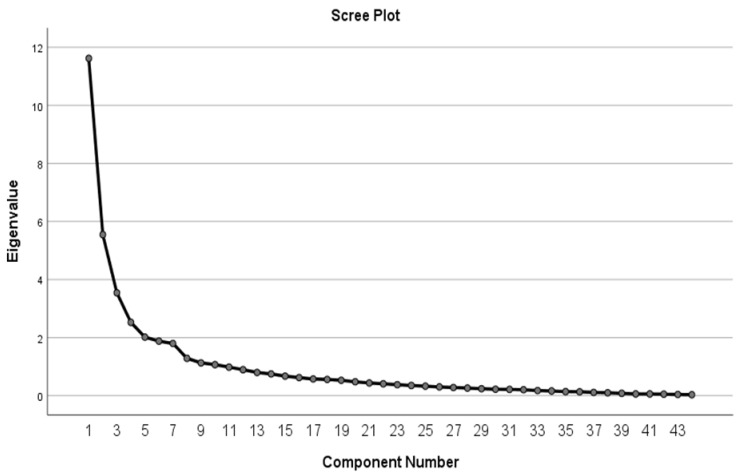
Scree plot test.

For the self-efficacy constructs, two factors with an eigenvalue of >1 and 76.8% CPV were extracted. The first and second component had a variance value of 55.5% and 21.3%, respectively. Two items (23, 26) were loaded on one component. The extracted perceived norms sub-constructs were labelled Factor 1, “intrinsic” and Factor 2, “extrinsic”. For the intention, behaviour, and barriers constructs, only one factor with an eigenvalue of >1 and 65.4%, 86.2%, and 63.6% CPV, respectively, was extracted. The scree plot test revealed seven factors with eigenvalues of >1 (Figure 3).

### 3.5. Reliability Testing

Table 5 shows that each construct extracted demonstrated different Cronbach’s alpha values: 0.855 for “perception implementation strategies of the AED” (five items), 0.637 for “community’s perception of the importance of CPR and AED” (three items), 0.637 for “community’s perception of AED handling training” (two items), 0.755 for “fearful of CPR and AED” (four items), 0.693 for “courage for CPR and AED” (three items), 0.633 for “perceived norms by relatives” (three items), 0.895 for “self-efficacy intrinsic” (three items), 0.878 for “intention” (six items), 0.945 for “behaviour” (four items), and 0.903 for “barriers” (seven items).

Examination of the construct “self-efficacy extrinsic” (two items) revealed that it had a corrected item–total correlation of <0.32 and Cronbach’s alpha of 0.111; therefore, it was eliminated [43]. The “attitude importance of CPR and AED” and “perceived norms community” components, which only had one item each, were also eliminated. This brought the total number of items deleted to four. After arrangement and deletion, there were a total of 40 items in the seven constructs. The corrected item–total correlation of all items was 0.378–0.897 and the overall Cronbach’s alpha, calculated at 0.849, indicated high reliability of the FIXED instrument. All constructs and sub-constructs yielded values of >0.60. According to DeVellis [42] and Straub et al. [47], Cronbach’s alpha reliability should be ≥0.60. This indicated that the FIXED instrument is reliable.

Data were collected from 45 participants using the same scale four weeks later (Table 6). The ICC values for the constructs and sub-constructs were as follows: AED perception implementation strategies, 0.932; community’s perception of the importance of CPR and AED, 0.643; community’s perception of AED handling training, 0.502; fearful of CPR and AED, 0.729; courage for CPR and AED, 0.746; perceived norms, 0.760; self-efficacy, 0.911; intention, 0.875; behaviours, 0.922; and barriers, 0.896. The overall ICC value was 0.723 with a 95% confidence interval of 0.614–0.819, indicating moderate to good reliability retests. The overall Cronbach’s alpha of 0.849 indicated that the FIXED questionnaire had high reliability.

## 4. Discussion

The FIXED questionnaire was developed to assess the factors that influence the community’s willingness to perform CPR and use an AED. Apart from its importance in determining the community’s willingness, this can empower the community by doing CPR and using an AED in emergency situations and can indirectly improve the survival of OHCA patients [48,49,50]. Although the questionnaire was developed with the community population as a primary target, the majority of items were not specifically related to the community, suggesting that the questionnaire may be useful in a variety of other populations upon further validation.

For the content validation, this study compared the I-CVI and the S-CVI, however, the majority of publications have reported either the I-CVI or the SCVI, but not both [51]. Both approaches can result in contradictory values, making it difficult to reach an accurate conclusion about content validity [25]. The content validity by five expert review panels achieved high content validity of I-CVI and high content validity of the overall questionnaire, whereby the items were appropriate for the study purpose. The proportion of agreement among the experts was considered to have excellent content validity (CVI = 0.98). This allows for easy interpretation of why the experts agreed that all original 59 items were appropriate and acceptable.

Following the review of the items by the expert panel, their responses were analysed using the FKI with the kappa value of 0.159, which indicated little agreement. This indicates the inability of the investigated measure or classification to clearly distinguish between subjects in a population where those distinctions are extremely rare or difficult to achieve [52,53]. Another possibility is that raters are unable to differentiate between adjacent categories [54]. The items were modified and improved in response to expert feedback, which included the use of simple words, revise medical terms to easy-to-understand terms, the avoidance of repetitive questions, and the rephrasing of sentences to the constructs’ relevance, adequateness, and representativeness.

For the construct validity testing, the KMO value for all seven constructs (perceptions, attitudes, perceived norms, self-efficacy, intentions, behaviours, barriers) and 44 items was 0.777. These findings are consistent with the findings of the original validation study by Chew et al. [20], which was 0.79. This may be influenced by the use of a validated construct from the original study for perceptions (perception of AED placement strategies and perception of importance of CPR and AED), barriers (concerns of injuring victims during CPR and AED, and concerns of legality in performing CPR and AED), and behaviours (confidence and willingness to perform CPR and AED), even though different populations produce nearly identical results. The KMO value for this study was between 0.7 and 0.8, indicating a good correlation with each other, and factor analysis was appropriate for this dataset. The Bartlett test of sphericity values for all constructs was significant, which is line with the previous study indicating that there was appropriate correlation between the items based on the correlation matrix [20]. The communality values for all items had a value of >0.25 (0.296–0.853). One item had a communality value of 0.4, that is, “knowing the importance of starting a resuscitation before EMS arrival” (0.296), which was considered unrelated to other items. However, according to Beavers et al. [55], communalities between 0.25 and 0.4 have been suggested as acceptable cut-off values, with ideal communalities being 0.7 or above.

All of the components were considered internally consistent, as each earned a Cronbach’s alpha value of >0.60, which coincides with the literature that the consistency of the construct using Cronbach’s alpha for a newly developed tool should be 0.60 [42,47]. Compared with the previous validation study by Chew et al. [20], the factor for “perception of AED placement strategies” was 0.942 compared to 0.915, showing the same excellent reliability results. The factor for “confidence and willingness to perform CPR and AED” was 0.893 compared to 0.945, showing an increase from high to excellent reliability results. However, the “perception of importance of CPR and AED” showed a significant difference from 0.855 (high reliability) to 0.637 (moderate reliability). This may be influenced by the participation of respondents in this study, who were only allocated a shorter time to learn CPR and AED compared to the original study involving participation in workshops, thus influencing their perceptions of the importance of CPR and AED at a moderate level. The internal consistency of the overall FIXED instrument was high reliability and the ICC indicated moderate to good test–retest reliability. Overall, the goods requirement in each construction as a whole met the KMO (>0.60), Bartlett’s test of sphericity (significant), factor loading exceeding the minimum threshold of 0.4, corrected item–total correlation >0.30, and the Cronbach’s alpha exceeded the minimum limit of 0.6 for adoption in this study.

The FIXED questionnaire translation and validation processes were difficult to compare with the previous study. There have been some limited validation studies even though they were for other languages. Some of the constructs from previous studies that were used in the FIXED questionnaire were not fully validated.

## 5. Conclusions

The FIXED instrument is the first tool for assessing the knowledge, training, perceptions, attitudes, perceived norms, self-efficacy, intentions, behaviours, and barriers within the community toward performing CPR and using an AED. The overall findings indicate that the Malay version of the instrument demonstrated acceptable validity and reliability in its pilot testing. In this evaluation, the validity and reliability of the instrument displayed the appropriate and acceptable measurement performance needed to assess the influencing factors affecting community willingness to perform CPR and use an AED in the Malaysian context. This tool has potential applications in both the research setting and clinical practice. Investigators can use it to survey their population of interest and use the information to inform decision-making to derive an effective strategy by implementing CPR- and AED-related comprehensive programmes involving the community. The tool can be tested on a larger sample to further establish its reliability and validity. In the future, the FIXED instrument is ready to be used by health care workers and policymakers to evaluate the factors influencing the community’s willingness to perform CPR and use an AED.

## Figures and Tables

**Table 1:** Socio-demographics characteristics of the participants (*n* = 100).

Characteristic	Frequency (*n*)	Percentage (%)
**Gender**		
(a) Male	51	51
(b) Female	49	49
**Age (years)**		
(a) below 40	80	80
(b) 40 and above	20	20
**Ethnicity**		
(a) Malay	77	77
(b) Chinese	15	15
(c) Indian	6	6
(d) Others	2 ^1^	2
**Level of education**		
(a) Secondary education	15	15
(b) Higher (Certificate/Diploma/Degree)	85	85
**Marital Status**		
(a) Single	44	44
(b) Married	53	53
(c) Widow/Widower	3	3
**Residential area**		
(a) City	61	61
(b) Rural	39	39
**Employment Status**		
(a) Health workers	42	42
(b) Employers	28	28
(c) Self-employed	8	8
(d) Students	15	15
(e) Pensions	5	5
(f) Housewives	1	1
(g) Not working	1	1
**Total household income (RM per month)**		
(a) B40 (below RM 4850) ^2^	59	59
(b) Not B40 (RM 4850 and above)	41	41
**Medical history of chronic illness**		
(a) Yes	3	3
(b) No	97	97
**Medical history of family members living together with chronic illnesses**		
(a) Yes	34	34
(b) No	66	66
**Experience of witnessing the incidence of cardiac arrest**		
(a) Ever	38	38
(b) Never	62	62
**Have performed CPR on a victim who suffered a cardiac arrest**		
(a) Ever	35	35
(b) Never	65	65

Footnotes: ^1^ Both from Dusun (Sabahan) ethnicity. ^2^ The B40 group was the Malaysia income classification and the range was below RM 4850, which equals USD 1145.

**Table 2:** Content validity index based on the construct to measure the factors influencing the community willingness to perform CPR and use an AED by five experts.

Item	Construct								
	1	2	3	4	5	6	7	8	9
**S-CVI/Ave**	1.0	1.0	1.0	0.88	0.95	1.0	1.0	1.0	1.0
**S-CVI/UA**	1.0	1.0	1.0	0.38	0.75	1.0	1.0	1.0	1.0
**Average proportion of items**	1.0	1.0	1.0	0.88	0.95	1.0	1.0	1.0	1.0

Note: Constructs: (1) knowledge, (2) training, (3) perceptions, (4) attitudes, (5) perceived norms, (6) self-efficacy, (7) intentions, (8) behaviours, and (9) barriers.

**Table 3:** Value of KMO and Bartlett’s test based on factor analysis.

Construct FIXED	Kaiser–Meyer–Olkin Measure of Sampling Adequacy	Bartlett’s Test of Sphericity		
		Approx. Chi-Square	df	Sig.
**FIXED Instrument**	**0.777**	**3345.614**	**946**	**<0.001**
Perception	0.802	493.299	45	<0.001
Attitude	0.643	203.192	28	<0.001
Perceived norms	0.582	48.267	6	<0.001
Self-efficacy	0.786	200.929	10	<0.001
Intention	0.854	370.516	15	<0.001
Behaviour	0.818	407.524	6	<0.001
Barriers	0.812	504.038	21	<0.001

**Table 4:** Factor analysis by PCA after varimax rotation for the perception, attitude, perceived norms, self-efficacy, intention, behaviour, and barriers construct.

Item No	Item	Factor Loading						
		Factor 1	Factor 2	Factor 3	Factor 4	Factor 5	Factor 6	Factor 7
**Perception**								
	**Implementation Strategies of the AED**							
2	The signage that shows the location of the AED is clear	0.922						
3	The AED is located in a location that is easily accessible at all times (including after office hours)	0.908						
4	The steps in the AED instructional poster on how to use the AED are easy to follow	0.853						
1	The AED is clearly visible	0.813						
5	The AED is located at a secure site	0.776						
	**Community’s perception on the importance of CPR and AED**							
6	CPR and AED are important in saving life	0.869						
7	It is important for an AED to be available in the place where I work.	0.851						
8	Using an AED is important on any unresponsive victims	0.457						
	**Community’s perception on the AED handling training**							
9	Person who handles an AED requires formal training.	0.840						
10	AED practice drills should be performed on a regular basis	0.817						
**Attitude**								
	**Fearful of CPR and AED**							
15	Not being afraid of worsening the victim’s condition		0.805					
16	Not being afraid of legal action		0.771					
14	Not being afraid of hurting the victim by performing CPR		0.768					
13	Not being afraid of disease transmission		0.682					
	**Courage for CPR and AED**							
18	Belief that knowing CPR is important for the society		0.871					
11	Thinking that performing resuscitation could save a life		0.811					
17	Being proud of performing resuscitation successfully		0.744					
	**Importance of CPR and AED**							
12	Knowing the importance of starting a resuscitation before EMS arrival		0.956					
**Perceived norms**								
	**Relatives**							
20	Belief that relatives want the subject to resuscitate them if needed			0.842				
21	Knowing that relatives are the most likely victim			0.774				
19	Belief that relatives would be proud if the participant performed resuscitation			0.661				
	**Community**							
22	Diffusion of responsibility			0.924				
**Self-efficacy**								
	**Intrinsic**							
24	Feeling able to resuscitate				0.904			
25	Feeling able to recognise a cardiac arrest				0.896			
27	Knowing how to perform a resuscitation				0.880			
	**Extrinsic**							
26	Not believing that only health care professionals can adequately perform resuscitation				0.787			
23	Knowledge of the emergency number				0.667			
**Intention**								
31	Perform CPR on an elderly person					0.901		
32	Perform CPR on a relative or family member					0.882		
28	Perform CPR on a stranger					0.869		
30	Perform CPR on a child					0.864		
33	Using an AED					0.692		
29	Perform CPR on a victim of trauma					0.593		
**Behaviour**								
37	Confident to use an AED on an unresponsive victim						0.942	
34	Confident to perform CPR						0.938	
35	Confident to use an AED						0.937	
36	Confident in recognising victim with no signs of life						0.897	
**Barriers**								
43	Concerned that I might be sued if I perform emergency CPR inappropriately							0.857
39	Concerned in injuring the victim when performing CPR							0.846
44	Concerned that I might be sued if I used an AED inappropriately							0.828
41	Concerned in injuring the victim if I use an AED device during CPR							0.819
40	Concerned in injuring myself when performing CPR							0.771
42	Concerned in injuring myself if I use an AED device during CPR							0.748
38	Concerned in getting infection from the victim when performing CPR							0.699

**Table 5:** Reliability of subscales.

Construct and Sub-Construct	Item	Corrected Item-Total Correlation	Cronbach’s Alpha before Deleting (44 Items)	Cronbach’s Alpha after Deleting (40 Items)
**Perception** **(Implementation Strategies of the AED)**	The signage that shows the location of the AED is clear	0.855	0.915	0.915
	The AED is located in a location that is easily accessible at all times (including after office hours)	0.835		
	The steps in the AED instructional poster on how to use the AED are easy to follow	0.790		
	The AED is clearly visible	0.713		
	The AED is located at a secure site	0.748		
**Perception** **(Community’s perception on the importance of CPR and AED)**	CPR & AED are important in saving life	0.482	0.637	0.637
	It is important for an AED to be available in the place where I work.	0.567		
	Using an AED is important on any unresponsive victims	0.378		
**Perception** **(Community’s perception on the AED handling training)**	Person who handles an AED requires formal training.	0.462	0.632	0.632
	AED practice drills should be performed on a regular basis	0.462		
**Attitude** **(Fearful of CPR and AED)**	Not being afraid of worsening the victim’s condition	0.617	0.755	0.755
	Not being afraid of legal action	0.568		
	Not being afraid of hurting the victim by performing CPR	0.589		
	Not being afraid of disease transmission	0.443		
**Attitude** **(Courage for CPR and AED)**	Belief that knowing CPR is important for the society	0.634	0.693	0.693
	Thinking that performing resuscitation could save a life	0.546		
	Being proud of performing resuscitation successfully	0.483		
**Attitude** **(Importance of CPR and AED)**	Knowing the importance of starting a resuscitation before EMS arrival			Component eliminated (one item)
**Perceived norms** **(Relatives)**	Belief that relatives want the subject to resuscitate them if needed	0.575	0.633	0.633
	Knowing that relatives are the most likely victim	0.395		
	Belief that relatives would be proud if the participant performed resuscitation	0.379		
**Perceived norms** **(Community)**	Diffusion of responsibility			Component eliminated (one item)
**Self-efficacy** **(Intrinsic)**	Feeling able to resuscitate	0.812	0.895	0.895
	Feeling able to recognise a cardiac arrest	0.799		
	Knowing how to perform a resuscitation	0.778		
**Self-efficacy** **(Extrinsic)**	Not believing that only health care professionals can adequately perform resuscitation	0.067	0.111	Component eliminated (two item)
	Knowledge of the emergency number	0.067		
**Intention**	Perform CPR on an elderly person	0.815	0.878	0.878
	Perform CPR on a relative or family member	0.785		
	Perform CPR on a stranger	0.772		
	Perform CPR on a child	0.761		
	Using an AED	0.586		
	Perform CPR on a victim of trauma	0.491		
**Behaviour**	Confident to use an AED on an unresponsive victim	0.897	0.945	0.945
	Confident to perform CPR	0.885		
	Confident to use an AED	0.887		
	Confident in recognising victim with no signs of life	0.818		
**Barriers**	Concerned that I might be sued if I perform emergency CPR inappropriately	0.786	0.903	0.903
	Concerned in injuring the victim when performing CPR	0.775		
	Concerned that I might be sued if I used an AED inappropriately	0.750		
	Concerned in injuring the victim if I use an AED device during CPR	0.735		
	Concerned in injuring myself when performing CPR	0.688		
	Concerned in injuring myself if I use an AED device during CPR	0.662		
	Concerned in getting infection from the victim when performing CPR	0.604		

**Table 6:** Reliability of FIXED based on internal consistency and test–retest re-liability.

Construct Dan Sub-Construct	ICC and 95% Confidence Interval		Cronbach’s Alpha	
	Construct	Overall	Construct	Construct
**Perception (** **Implementation Strategies of the AED)**	0.932 (0.898–0.958)	0.723 (0.614–0.819)	0.915	0.849
**Perception (** **Community’s perception on the importance of** **CPR and AED** **)**	0.643 (0.457–0.782)		0.637	
**Perception (** **Community’s perception on the AED handling training)**	0.502 (0.216–0.702)		0.632	
**Attitude (Fearful of CPR and AED)**	0.729 (0.585–0.835)		0.755	
**Attitude (Courage for CPR and AED)**	0.746 (0.608–0.846)		0.693	
**Perceived norms (Relatives)**	0.760 (0.635–0.854)		0.633	
**Self-efficacy** **(Intrinsic)**	0.911 (0.863–0.946)		0.895	
**Intention**	0.875 (0.813–0.922)		0.878	
**Behaviour**	0.922 (0.881–0.952)		0.945	
**Barriers**	0.896 (0.845–0.935)		0.903	

## Data Availability

The data presented in this study are available within the article.

## Supplementary Materials

The following supporting information can be downloaded at: https://www.mdpi.com/article/10.3390/ijerph19084882/s1. Supplementary Materials Table S1. A written report recording all synthesis processes; Supplementary Materials Table S2. Summarises the information of the content validity expert review panel of this instrument; Supplementary Materials Table S3. Conceptual and operational definitions of FIXED; Supplementary Materials Table S4. The relevance ratings on the item scale by five experts; Supplementary Materials Table S5. The Fleiss Kappa index based on a suitability level assessment by five experts; Supplementary Materials Table S6. Communalities values for all items.
