# Supplementary material for: Translation, Cross-Cultural Adaptation, and Validation of the Malay-Version of the Factors Influencing Community Willingness to Perform Cardiopulmonary Resuscitation and Use an Automated External Defibrillator Questionnaire
Source: Int J Environ Res Public Health. 2022 Apr 17;19(8):4882. doi: 10.3390/ijerph19084882 (PMC9028418; doi:10.3390/ijerph19084882)
Supplement: Supplementary file 1 [file ijerph-19-04882-s001.zip › ijerph-1654744-supplementary.pdf]

SUPPLEMENTARY MATERIAL

Supplementary Material Table S1. A written report recording all synthesis processes

SYNTHESIS PROCESS 1: ORIGINAL ENGLISH TO MALAY LANGUAGE (FORWARD TRANSLATION)

Construct	Original English version	Mrs. T1	Mrs. T2	Conciliation
**Knowledge**	Can CPR be performed outside a hospital setting?<br>a) Yes<br>b) No<br>c) Not sure	Adakah CPR boleh dilakukan di luar situasi hospital?<br>a) Ya<br>b) Tidak<br>c) Tidak Pasti	Bolehkah CPR dilakukan di luar kawasan hospital?<br>a) Ya<br>b) Tidak<br>c) Tidak Pasti	Bolehkah CPR dilakukan di luar kawasan hospital?<br>a) Ya<br>b) Tidak<br>c) Tidak Pasti
	Can bystander CPR* be performed without mouth-to-mouth resuscitation?<br>**Bystander CPR; CPR that is performed by a layperson who is not part of organized emergency-response system in a community.*<br>a) Yes<br>b) No<br>c) Not sure	Adakah 'bystander CPR'* boleh dilakukan tanpa bantuan pernafasan mulut-ke-mulut?<br>**Bystander CPR; CPR yang dilakukan oleh mereka yang berada di situ dan bukan sebahagian daripada pasukan bantuan kecemasan komuniti*<br>a) Ya<br>b) Tidak<br>c) Tidak Pasti	Bolehkah CPR oleh saksi kejadian dilakukan tanpa bantuan pernafasan mulut ke mulut?<br>**CPR oleh saksi kejadian; CPR yang dilakukan oleh orang awam yang bukan daripada badan sistem kecemasan dalam komuniti.*<br>a) Ya<br>b) Tidak<br>c) Tidak Pasti	Bolehkah CPR oleh orang awam di tempat kejadian dilakukan tanpa bantuan pernafasan mulut ke mulut?<br>**CPR oleh orang awam di tempat kejadian; CPR yang dilakukan oleh orang awam yang bukan daripada badan sistem kecemasan dalam komuniti.*<br>a) Ya<br>b) Tidak<br>c) Tidak Pasti
	Can a person perform CPR without certification?<br>a) Yes<br>b) No<br>c) Not sure	Adakah seseorang itu boleh melakukan CPR tanpa sebarang perakuan?<br>a) Ya<br>b) Tidak<br>c) Tidak Pasti	Bolehkah seseorang individu melakukan CPR tanpa sebarang pensijilan?<br>a) Ya<br>b) Tidak<br>c) Tidak Pasti	Bolehkah seseorang individu melakukan CPR tanpa sebarang pensijilan?<br>a) Ya<br>b) Tidak<br>c) Tidak Pasti
	What is the correct sequence to perform CPR? *<br>**Airway; Make sure the airway is clear. Breathing; Look, listen and feel for signs*	Apakah susunan yang betul untuk melakukan CPR?*<br>**Saluran Pernafasan; Memastikan saluran pernafasan lancar.*	Apakah susunan yang betul dalam melakukan CPR?*<br>**Saluran udara; Pastikan saluran udara kosong. Pernafasan; Lihat, dengar dan*	Apakah susunan yang betul dalam melakukan CPR?)*<br>**Saluran Pernafasan; Memastikan saluran pernafasan lancar.*

	of breathing. Circulation; Perform a chest compression to provide adequate blood flow to the body<br>a) Circulation > Airway > Breathing<br>b) Airway > Breathing > Circulation<br>c) Not sure	*Pernafasan; Tengok,dengar dan rasa untuk sebarang tanda bernafas. Peredaran; Melakukan penekanan di bahagian dada untuk membenarkan peredaran darah yang lebih baik ke seluruh badan*<br>a) Peredaran > Saluran Pernafasan > Pernafasan<br>b) Saluran Pernafasan > Pernafasan > Peredaran<br>c) Tidak pasti	*cari tanda-tanda pernafasan. Pengedaran; Lakukan kompresi dada untuk memastikan peredaran darah yang mencukupi dalam badan.*<br>a) Pengedaran > Saluran udara > Pernafasan<br>b) Saluran udara > Pernafasan > Pengedaran<br>c) Tidak pasti	*Pernafasan; Tengok,dengar dan rasa untuk sebarang tanda bernafas. Peredaran; Melakukan penekanan di bahagian dada untuk membenarkan peredaran darah yang lebih baik ke seluruh badan*<br>a) Peredaran > Saluran Pernafasan > Pernafasan<br>b) Saluran Pernafasan > Pernafasan > Peredaran<br>c) Tidak pasti
	When should one perform CPR? (You may tick more than one option)<br>a) When a person is unconscious<br>b) When a person is not breathing<br>c) When a person complains of chest pain<br>d) When there is no pulse felt<br>e) When a person looks pale/blue<br>f) When a person complains of feeling dizzy<br>g) Not sure	Bilakah waktu yang sesuai untuk seseorang melakukan CPR? (And boleh pilih lebih daripada satu)<br>a) Apabila seseorang itu tidak sedarkan diri<br>b) Apabila seseorang itu tidak bernafas<br>c) Apabila seseorang itu mengadu sakit dada<br>d) Apabila tiada denyutan nadi yang boleh dirasa<br>e) Apabila seseorang itu kelihatan pucat/muka kebiruan<br>f) Apabila sesorang itu mengadu berasa pening<br>g) Tidak pasti	Bilakah seseorang individu perlu melakukan CPR? (Boleh tanda lebih daripada satu pilihan jawapan)<br>a) Apabila seseorang tidak menyedarkan diri.<br>b) Apabila seseorang tidak bernafas.<br>c) Apabila seseorang mengadu sakit dada<br>d) Apabila tiada nadi dapat dikesan<br>e) Apabila seseorang kelihatan pucat/kebiruan<br>f) Apabila seseorang mengadu berasa pening kepala<br>g) Tidak pasti	Bilakah seseorang individu perlu melakukan CPR? (Boleh tanda lebih daripada satu pilihan jawapan)<br>a) Apabila seseorang itu tidak sedarkan diri<br>b) Apabila seseorang itu tidak bernafas.<br>c) Apabila seseorang itu mengadu sakit dada<br>d) Apabila tiada nadi dapat dikesan<br>e) Apabila seseorang kelihatan pucat/kebiruan<br>f) Apabila seseorang mengadu berasa pening kepala<br>g) Tidak pasti

	Where is the right location to perform chest compression?<br>a) A<br>b) B<br>c) C<br>d) D<br>e) Not sure	Di manakah tempat yang sesuai untuk melakukan penekanan dada?<br>a) A<br>b) B<br>c) C<br>d) D<br>e) Tidak pasti	Dimanakah bahagian yang tepat untuk melakukan kompresi dada?<br>a) A<br>b) B<br>c) C<br>d) D<br>e) Tidak pasti	Dimanakah bahagian yang tepat untuk melakukan penekanan dada?<br>a) A<br>b) B<br>c) C<br>d) D<br>e) Tidak pasti
	How fast should one perform chest compression?<br>a) 150 compressions per minute<br>b) 100 compressions per minute<br>c) 50 compressions per minute<br>d) As fast as possible<br>e) Not sure<br>f) Others (please specify)	Berapa lajukah seseorang boleh melakukan penekanan dada?<br>a) 150 tekanan seminit<br>b) 100 tekanan seminit<br>c) 50 tekanan seminit<br>d) Selaju yang mungkin<br>e) Tidak pasti<br>f) Lain-lain (Sila nyatakan)	Seberapa lajukah seseorang perlu melakukan kompresi dada?<br>a) 150 kompresi per minit<br>b) 100 kompresi per minit<br>c) 50 kompesi per minit<br>d) Seberapa cepat yang mungkin<br>e) Tidak pasti<br>f) Lain-lain (sila nyatakan)	Berapa lajukah seseorang boleh melakukan penekanan dada?<br>a) 150 tekanan seminit<br>b) 100 tekanan seminit<br>c) 50 tekanan seminit<br>d) Selaju yang mungkin<br>e) Tidak pasti<br>f) Lain-lain (Sila nyatakan)
	What is the correct depth of compression that should be performed during chest compression?<br>a) Such that the rib cage moves down 1 to 2 cm<br>b) Such that the rib cage moves down 5 to 6 cm<br>c) Such that the rib cage moves down 6 to 10 cm<br>d) As deep as possible<br>e) Not sure<br>f) Others (please specify)	Apakah kedalaman untuk tekanan yang betul ketika penekanan dada dilakukan?<br>a) Sehingga tulang rusuk turun ke bawah 1 hingga 2 cm<br>b) Sehingga tulang rusuk turun ke bawah 5 hingga 6 cm<br>c) Sehingga tulang rusuk turun ke bawah 6 hingga 10 cm<br>d) Sedalam yang mungkin<br>e) Tidak pasti<br>f) Lain-lain (Sila nyatakan)	Apakah kedalaman kompresi yang betul yang perlu dilakukan semasa proses kompresi dada?<br>a) Sehingga sangkar tulang rusuk bergerak ke bawah 1 ke 2 cm.<br>b) Sehingga sangkar tulang rusuk bergerak ke bawah 5 ke 6 cm.<br>c) Sehingga sangkar tulang rusuk bergerak ke bawah 6 ke 10 cm.<br>d) Sedalam yang boleh<br>e) Tidak pasti	Apakah kedalaman yang betul ketika penekanan dada dilakukan?<br>a) Sehingga tulang rusuk turun ke bawah 1 hingga 2 cm<br>b) Sehingga tulang rusuk turun ke bawah 5 hingga 6 cm<br>c) Sehingga tulang rusuk turun ke bawah 6 hingga 10 cm<br>d) Sedalam yang mungkin<br>e) Tidak pasti<br>f) Lain-lain (Sila nyatakan)

			f) Lain-lain (sila nyatakan)	
	Have you ever heard of AED?<br>a) Yes<br>b) No	Adakah anda pernah dengar tentang AED?<br>a) Ya<br>b) Tidak	Pernahkah anda dengar tentang AED?<br>a) Ya<br>b) Tidak	Pernahkah anda dengar tentang AED?<br>a) Ya<br>b) Tidak
	Do you know that prompt use of an AED can increase survival?<br>a) Yes<br>b) No	Adakah anda tahu penggunaan AED yang serta-merta boleh meningkatkan kebarangkalian untuk selamat?<br>a) Ya<br>b) Tidak	Adakah anda tahu bahawa melakukan AED dengan segera dapat meningkatkan peluang hidup?<br>a) Ya<br>b) Tidak	Adakah anda tahu bahawa melakukan AED dengan segera dapat meningkatkan peluang hidup?<br>a) Ya<br>b) Tidak
	Do you know the location of an AED nearest to your home or workplace?<br>a) Yes<br>b) No	Adakah anda tahu lokasi terdekat AED ke tempat tinggal atau tempat kerja anda?<br>a) Ya<br>b) Tidak	Adakah anda tahu lokasi AED berdekatan dengan rumah atau tempat kerja anda?<br>a) Ya<br>b) Tidak	Adakah anda tahu lokasi AED berdekatan dengan rumah atau tempat kerja anda?<br>a) Ya<br>b) Tidak
	What is the correct position for placement of the AED pads?<br>a) A-A<br>b) B-B	Di manakah tempat yang betul untk meletakkan pad AED?<br>a) A-A<br>b) B-B	Apakah posisi yang betul untuk meletakkan pad AED?<br>a) A-A<br>b) B-B	Dimanakah bahagian yang betul untuk meletakkan pad AED?<br>a) A-A<br>b) B-B
	Do you know there is voice prompt in the AED?<br>a) Yes<br>b) No	Adakah anda tahu tentang bantuan suara yang terdapat di AED?<br>a) Ya<br>b) Tidak	Adakah anda tahu terdapat arahan bersuara di dalam AED?<br>a) Ya<br>b) Tidak	Adakah anda tahu terdapat arahan bersuara di dalam AED?<br>a) Ya<br>b) Tidak
**Training**	Total no. of CPR education sessions attended	Jumlah sesi latihan CPR yang diikuti	Jumlah sesi pembelajaran CPR dihadiri	Jumlah sesi pembelajaran CPR dihadiri
	Period from the last CPR education session	Jangka masa sejak sesi latihan CPR yang terakhir	Jarak masa daripada sesi pembelajaran CPR terakhir	Jarak masa daripada sesi pembelajaran CPR terakhir
**Attitude**	Performing a resuscitation would enable me to save a life	Melakukan bantuan pernafasan membantu saya menyelamatkan nyawa	Melakukan bantuan pernafasan dapat membantu saya menyelamatkan nyawa.	Melakukan resusitasi/bantuan pernafasan dapat membantu saya menyelamatkan nyawa.

	If I call for help for someone in cardiac arrest when I am in a big city (where an ambulance comes quickly), the victim's chances of survival will not be altered whether I perform resuscitation or not.	Jika saya meminta bantuan untuk seseorang yang mengalami serangan jantung ketika saya berada di sebuah bandar besar (di mana ambulans akan tiba dengan segera), kadar kebarangkalian manga untuk selamat tidak berubah sama ada saya melakukan bantuan pernafasan atau tidak.	Jika saya memerlukan bantuan pernafasan untuk seseorang yang menghadapi jantung berhenti dengan mengejut semasa saya di dalam bandar besar (dimana ambulans dapat datang dengan segera), peluang mangsa untuk hidup tidak akan berubah jika saya melakukan bantuan pernafasan atau tidak.	Jika saya meminta bantuan untuk seseorang yang mengalami serangan jantung ketika saya berada di sebuah bandar besar (dimana ambulans dapat datang dengan segera), peluang mangsa untuk hidup tidak akan berubah jika saya melakukan bantuan pernafasan atau tidak.
	By resuscitating a stranger, I can catch diseases (for example AIDS or hepatitis)	Saya boleh jika dijangkiti pelbagai penyakit apabila memberi bantuan pernafasan kepada orang yang tidak dikenali (contoh: AIDS or hepatitis)	Dengan memberi bantuan pernafasan terhadap orang yang tidak dikenali, saya berisiko untuk mendapat penyakit (sebagai contoh AIDS atau hepatitis)	Dengan memberi bantuan pernafasan terhadap orang yang tidak dikenali, saya berisiko untuk mendapat penyakit (sebagai contoh AIDS atau hepatitis)
	By performing chest compressions on a cardiac arrest victim, I might injure him even more (for example by breaking his ribs which can pierce his lung). This can be life threatening. It is therefore better not to attempt resuscitation and call for help.	Apabila melakukan penekanan dada kepada orang yang mengalami serangan jantung, saya mungkin akan mencederakan dia (Contoh: mematahkan tulang rusuk dan tertusuk paru-paru). Ini boleh membahayakan nyawa, maka lebih baik untuk saya tidak melakukan bantuan pernafasan dan memanggil untuk bantuan.	Dengan melakukan kompresi kepada mangsa yang menghadapi jantung berhenti mengejut, saya mungkin akan lebih menyakitinya (contohnya mematahkan tulang rusuk dengan tidak sengaja yang boleh menyebabkan kecederaan kepada paru-paru). Ini boleh membahayakan nyawa. Jadi adalah lebih baik untuk tidak melakukan bantuan pernafasan dan terus dapatkan pertolongan.	Dengan melakukan penekanan dada kepada mangsa yang mengalami serangan jantung, saya mungkin akan lebih mencederakan dia (contohnya mematahkan tulang rusuk dengan tidak sengaja yang boleh menyebabkan kecederaan kepada paru-paru). Ini boleh membahayakan nyawa. Jadi adalah lebih baik untuk saya tidak melakukan bantuan pernafasan dan terus dapatkan pertolongan.
	If one does not exactly know how to provide chest compressions but finds an unconscious person who is	Jika seseorang tidak pasti cara yang betul untuk melakukan penekanan dada dan terjumpa seseorang yang pengsan dan	Jika seseorang tidak berapa tahu bagaimana untuk melakukan kompresi dada tetapi berhadapan dengan	Jika seseorang tidak pasti cara yang betul untuk melakukan penekanan dada tetapi terjumpa seseorang yang tidak

	not breathing, it is better to wait for the ambulance than initiate resuscitation. One would risk hurting the person by performing the resuscitation maneuvers in a wrong way.	tidak bernafas, adalah lebih baik untuk menunggu ambulans daripada memberikan bantuan pernafasan. Dia boleh mencederakan mangsa jika melakukan cara bantuan pernafasan yang salah.	orang yang sedang tidak bernafas, adalah lebih baik untuk menunggu ambulan berbanding melakukan bantuan pernafasan terhadapnya. Seseorang itu akan memberi risiko kepada mangsa jika melakukan bantuan pernafasan dengan cara yang salah.	sedarkan diri dan tidak bernafas, adalah lebih baik untuk menunggu ambulans daripada memulakan resusitasi/bantuan pernafasan. Dia boleh mencederakan mangsa jika melakukan resusitasi/bantuan pernafasan dengan cara yang salah.
	If I perform resuscitation and injure the victim, I could have problems with the law.	Jika saya melakukan bantuan pernafasan dan mencederakan mangsa, saya akan mengalami masalah dengan undang-undang.	Jika saya melakukan bantuan pernafasan dan kemudiannya memberi kesakitan kepada pesakit, saya boleh dikenakan tindakan undang-undang.	Jika saya melakukan bantuan pernafasan dan mencederakan mangsa, saya boleh dikenakan tindakan undang-undang.
	I would be proud to have resuscitated someone.	Saya akan berasa bangga apabila dapat menyedarkan seseorang.	Saya rasa bangga jika dapat memberi bantuan pernafasan kepada seseorang.	Saya akan berasa bangga apabila dapat memberi bantuan pernafasan kepada seseorang.
	Being able to perform resuscitation is a good thing for the society.	Dapat melakukan bantuan pernafasan adalah perkara yang baik untuk masyarakat.	Dapat memberi bantuan pernafasan adalah sesuatu yang bagus dalam kalangan sosial.	Dapat melakukan bantuan pernafasan adalah perkara yang baik untuk masyarakat.
**Perceived Norms**	My family and friends would be proud if I performed a resuscitation.	Ahli keluarga dan kawan saya akan berasa bangga jika saya melakukan bantuan pernafasan.	Saya dan keluarga akan berasa bangga jika saya melakukan bantuan pernafasan.	Ahli keluarga dan kawan saya akan berasa bangga jika saya melakukan bantuan pernafasan.
	If a family member or a friend had a cardiac arrest, he would surely want me to try to resuscitate him in an attempt to save him.	Jika ahli keluarga atau kawan mengalami serangan jantung, mereka akan mahukan saya untuk melakukan bantuan pernafasan untuk cuba menyelamatkan mereka.	Jika ada ahli keluarga atau rakan yang menghadapi jantung berhenti mengejut, individu tersebut pasti mahukan saya untuk cuba melakukan bantuan pernafasan dan menyelamatkan hidupnya.	Jika ada ahli keluarga atau kawan mengalami serangan jantung, mereka akan mahukan saya untuk melakukan bantuan pernafasan untuk cuba menyelamatkan mereka.
	If I witness a cardiac arrest someday, the victim would most likely (statistically	Jika saya ternampak serangan jantung, mangsa kemungkinan besar (mengikut statisitk)	Jika suatu hari saya menyaksikan situasi jantung berhenti mengejut, mangsa	Jika suatu hari saya menyaksikan serangan jantung, mangsa tersebut

	speaking) be a relative (family member or friend)	adalah seseorang yang saya kenal (ahli keluarga atau kawan)	tersebut berkemungkinan besar (secara statistik) adalah dalam kalangan saudara terdekat (keluarga atau rakan).	berkemungkinan besar (secara statistik) adalah dalam kalangan saudara terdekat (ahli keluarga atau rakan).
	If I'm in a crowded train station and someone collapses in front of me, I should better wait and see if someone better trained in resuscitation maneuvers is present before taking action.	Jika saya berada di dalam stesen kereta api yang sesak dan seseorang jatuh pengsan di depan saya, saya akan tunggu jika ada orang lain yang lebih terlatih dalam bantuan pernafasan sebelum mengambil tindakan.	Jika saya berada didalam stesen keretapi yang sesak dan seseorang rebah dihadapan saya, adalah lebih baik jika saya tunggu seseorang yang terlatih dalam memberi bantuan pernafasan untuk datang membantu sebelum saya melakukan tindakan.	Jika saya berada di dalam stesen kereta api yang sesak dan seseorang jatuh pengsan dihadapan saya, adalah lebih baik saya tunggu dan lihat jika seseorang yang lebih terlatih dalam memberi resusistasi/bantuan pernafasan sebelum saya melakukan tindakan.
**Self -Efficacy**	Know the call number of the ambulance in Malaysia. Therefore, I could quickly raise the alarm if I found an unconscious person who was not breathing.	Ingat nombor ambulans di Malaysia. Saya boleh terus meminta bantuan jika terjumpa seseorang pengsan dan tidak bernafas.	Sedia tahu nombor panggilan kecemasan ambulan. Dengan itu, saya dapat melakukan tindakan segera jika saya berhadapan dengan seseorang yang tidak bernafas.	Sedia tahu nombor panggilan ambulan di Malaysia. Dengan itu, saya dapat melakukan tindakan segera jika saya berhadapan dengan seseorang yang tidak sedarkan diri dan tidak bernafas.
	I feel able to perform resuscitation.	Saya rasa mampu untuk melakukan bantuan pernafasan.	Saya boleh melakukan bantuan pernafasan.	Saya rasa saya boleh melakukan resusitasi/bantuan pernafasan.
	I can recognize someone who needs resuscitation.	Saya boleh mengenalpasti jika seseorang memerlukan bantuan pernafasan.	Saya dapat mengenalpasti individu yang memerlukan bantuan pernafasan.	Saya boleh mengenalpasti jika seseorang memerlukan bantuan pernafasan.
	Only a health care professional can perform a truly effective resuscitation.	Hanya ahli rawatan professional yang boleh melakukan bantuan pernafasan yang berkesan.	Hanya pakar kesihatan boleh melakukan bantuan pernafasan yang betul-betul efektif.	Hanya professional kesihatan yang boleh melakukan resusitasi/bantuan pernafasan yang berkesan.
	I know how to perform resuscitation maneuvers: I could therefore help a victim of cardiac arrest efficiently.	Saya tahu cara untuk melakukan bantuan pernafasan yang betul: Dengan itu saya boleh membantu mangsa	Saya tahu bagaimana untuk melakukan bantuan pernafasan. Jadi saya boleh bantu mangsa yang	Saya tahu bagaimana cara untuk melakukan bantuan pernafasan. Dengan itu saya boleh membantu mangsa

		serangan jantung dengan berkesan.	menghadapi jantung berhenti mengejut secara efisyen.	serangan jantung dengan efisyen.
**Intention**	In an emergency situation, would you perform CPR on a stranger?	Dalam keadaan kecemasan, adakah anda akan melakukan CPR kepada orang yang tidak dikenali?	Dalam situasi kecemasan, adakah anda akan melakukan CPR kepada orang yang tidak dikenali?	Dalam situasi kecemasan, adakah anda akan melakukan CPR kepada orang yang tidak dikenali?
	In an emergency situation, would you perform CPR on a victim of trauma?	Dalam keadaan kecemasan, adakah anda akan melakukan CPR kepada mangsa trauma?	Dalam situasi kecemasan, adakah anda akan melakukan CPR kepada mangsa trauma?	Dalam situasi kecemasan, adakah anda akan melakukan CPR kepada mangsa trauma?
	In an emergency situation, would you perform CPR on a child?	Dalam keadaan kecemasan, adakah anda akan melakukan CPR kepada seorang kanak-kanak?	Dalam situasi kecemasan, adakah anda akan melakukan CPR kepada kanak-kanak?	Dalam situasi kecemasan, adakah anda akan melakukan CPR kepada kanak-kanak?
	In an emergency situation, would you perform CPR on an elderly person?	Dalam keadaan kecemasan, adakah anda akan melakukan CPR kepada seorang yang berusia?	Dalam situasi kecemasan, adakah anda akan melakukan CPR kepada orang tua?	Dalam situasi kecemasan, adakah anda akan melakukan CPR kepada orang tua?
	In an emergency situation, would you perform CPR on a relative or family member?	Dalam keadaan kecemasan, adakah anda akan melakukan CPR kepada kenalan atau ahli keluarga?	Dalam situasi kecemasan, adakah anda akan melakukan CPR kepada saudara atau keluarga?	Dalam situasi kecemasan, adakah anda akan melakukan CPR kepada saudara atau ahli keluarga?
	In an emergency situation, would you use an AED?	Dalam keadaan kecemasan, adakah anda akan menggunakan AED?	Dalam situasi kecemasan, adakah anda akan menggunakan AED?	Dalam situasi kecemasan, adakah anda akan menggunakan AED?

*The red highlight colour represents the discrepancies and issues addressed between translators

*The green highlight colour represents the synthesis of intensive discussions among the experts and translators

SYNTHESIS PROCESS 2 : MALAY LANGUAGE TO ENGLISH (BACKWARD TRANSLATION)

Construct	Conciliation	Mrs. B1	Mrs. B4	Harmonising
**Knowledge**	Bolehkah CPR dilakukan di luar kawasan hospital?<br>a) Ya<br>b) Tidak<br>c) Tidak Pasti	Can CPR be conducted outside the hospital compound?<br>a) Yes<br>b) No<br>c) Not sure	Can CPR be done outside of hospital compound?<br>a) Yes<br>b) No<br>c) Not sure	Can CPR be conducted outside the hospital compound?<br>a) Yes<br>b) No<br>c) Not sure
	Bolehkah CPR oleh orang awam di tempat kejadian dilakukan tanpa bantuan pernafasan mulut ke mulut?<br>**CPR oleh orang awam* di tempat kejadian; *CPR yang dilakukan oleh orang awam yang bukan daripada badan sistem kecemasan dalam komuniti.*<br>a) Ya<br>b) Tidak<br>c) Tidak Pasti	Can CPR be conducted by any incident witness without mouth-to-mouth resuscitation?<br>a) Yes<br>b) No<br>c) Not sure	Can CPR done by any incident witness be allowed without mouth-to-mouth breathing assistance?<br>* CPR is done by the incident witness; CPR is performed by individuals who are not from emergency system of the community<br>a) Yes<br>b) No<br>c) Not sure	Can CPR done by any incident witness be allowed without mouth-to-mouth breathing assistance?<br>** CPR is done by the incident witness; CPR is performed by individuals who are not from emergency system of the community*<br>a) Yes<br>b) No<br>c) Not sure
	Bolehkah seseorang individu melakukan CPR tanpa sebarang pensijilan?<br>a) Ya<br>b) Tidak<br>c) Tidak Pasti	Can an individual perform CPR without any certification<br>a) Yes<br>b) No<br>c) Not sure	Can an individual perform CPR without any certification?<br>a) Yes<br>b) No<br>c) Not sure	Can an individual perform CPR without any certification?<br>a) Yes<br>b) No<br>c) Not sure
	Apakah susunan yang betul dalam melakukan CPR?)*<br>**Saluran Pernafasan; Memastikan saluran pernafasan lancar. Pernafasan; Tengok,dengar dan rasa untuk sebarang*	What is the correct order of performing CPR?<br>**Respiratory Airway : Ensure the airway is smooth. Breathing; look, listen and feel for any sign of breathing. Circulation; Practice*	What are the correct steps in performing CPR?<br>*Breathing airway; make sure the airway is clear. Resuscitation; observe, listen and feel for any breathing signs. Circulation;*	What are the correct steps in performing CPR? *<br>*Breathing airway; make sure the airway is clear. Breathing; look, listen and feel for any sign of breathing. Circulation; performing chest*

	tanda bernafas. Peredaran; Melakukan penekanan di bahagian dada untuk membenarkan peredaran darah yang lebih baik ke seluruh badan<br>a) Peredaran > Saluran Pernafasan > Pernafasan<br>b) Saluran Pernafasan > Pernafasan > Peredaran<br>c) Tidak pasti	*compression on the chest area to allow better blood circulation to entire body.*<br>a) Circulation >Respiratory Airway> Breathing<br>b) Respiratory Airway> Breathing > Circulation<br>c) Not sure	*performing chest compression to allow better blood circulation to the whole body.*<br>a) Circulation > Breathing airway > Resuscitation<br>b) Breathing airway > Resuscitation > Circulation<br>c) Not sure	*compression to allow better blood circulation to the whole body.*<br>a) Circulation > Breathing airway > Breathing<br>b) Breathing airway > Breathing > Circulation<br>c) Not sure
	Bilakah seseorang individu perlu melakukan CPR? (Boleh tanda lebih daripada satu pilihan jawapan)<br>a) Apabila seseorang itu tidak sedarkan diri<br>b) Apabila seseorang itu tidak bernafas.<br>c) Apabila seseorang itu mengadu sakit dada<br>d) Apabila tiada nadi dapat dikesan<br>e) Apabila seseorang kelihatan pucat/kebiruan<br>f) Apabila seseorang mengadu berasa pening kepala<br>g) Tidak pasti	When an individual is supposed to perform CPR? (You may choose more than one answer)<br>a) When a person is unconscious<br>b) When a person is not breathing<br>c) When a person complains of chest pain<br>d) When no pulse can be detected<br>e) When a person looks pale or bluish.<br>f) When a person complains of dizziness<br>g) Not sure	When does one need to perform CPR? (Please select all that apply)<br>a) When one is unconcious<br>b) When one does not breathe<br>c) When one has chest pain<br>d) When one has no pulse<br>e) When one looks pale or bluish<br>f) When one has headache<br>g) Not sure	When an individual is supposed to perform CPR? (You may choose more than one answer)<br>a) When a person is unconscious<br>b) When a person is not breathing<br>c) When a person complains of chest pain<br>d) When no pulse can be detected<br>e) When a person looks pale or bluish.<br>f) When a person complains of dizziness<br>g) Not sure
	Dimanakah bahagian yang tepat untuk melakukan penekanan dada?<br>a) A<br>b) B<br>c) C	Which one is the exact area to do chest compression?<br>a) A<br>b) B<br>c) C<br>d) D	Where is the exact area to perform chest compression?<br>a) A<br>b) B<br>c) C<br>d) D	Where is the exact area to perform chest compression?<br>a) A<br>b) B<br>c) C<br>d) D

	d) D<br>e) Tidak pasti	e) Not sure	e) Not sure	e) Not sure
	Berapa lajukah seseorang boleh melakukan penekanan dada?<br>a) 150 tekanan seminit<br>b) 100 tekanan seminit<br>c) 50 tekanan seminit<br>d) Selaju yang mungkin<br>e) Tidak pasti<br>f) Lain-lain (Sila nyatakan)	How fast a person can do chest compression?<br>a) 150 compressions per minute<br>b) 100 compressions per minute<br>c) 50 compressions per minute<br>d) As fast as possible<br>e) Not sure<br>f) Others (Please State)	How fast does one can perform chest compression?<br>a) 150 compressions per minute<br>b) 100 compressions per minute<br>c) 50 compressions per minute<br>d) As fast as one could<br>e) Not sure<br>g) f) Others (Please state)	How fast does one can perform chest compression?<br>a) 150 compressions per minute<br>b) 100 compressions per minute<br>c) 50 compressions per minute<br>d) As fast as possible<br>e) Not sure<br>f) Others (Please state)
	Apakah kedalaman yang betul ketika penekanan dada dilakukan?<br>a) Sehingga tulang rusuk turun ke bawah 1 hingga 2 cm<br>b) Sehingga tulang rusuk turun ke bawah 5 hingga 6 cm<br>c) Sehingga tulang rusuk turun ke bawah 6 hingga 10 cm<br>d) Sedalam yang mungkin<br>e) Tidak pasti<br>f) Lain-lain (Sila nyatakan)	What is the depth for the correct pressure when chest compressions are performed?<br>a) Until the ribs alight to 1 and 2 cm<br>b) Until the ribs alight to 5 and 6 cm<br>c) Until the ribs alight to 6 and 10 cm<br>d) As deep as possible<br>e) Not sure<br>f) Others (Please State)	What is the accurate compression depth while performing chest compression?<br>a) Until the rib comes to rest between 1 to 2 cm<br>b) Until the rib comes to rest between 5 to 6 cm<br>c) Until the rib comes to rest between 6 to 10 cm<br>d) As deep as possible<br>e) Not sure<br>f) Others (Please state)	What is the accurate compression depth while performing chest compression?<br>a) Until the ribs alight to 1 and 2 cm<br>b) Until the ribs alight to 5 and 6 cm<br>c) Until the ribs alight to 6 and 10 cm<br>d) As deep as possible<br>e) Not sure<br>f) Others (Please State)
	Pernahkah anda dengar tentang AED?<br>a) Ya<br>b) Tidak	Have you ever heard AED?<br>a) Yes<br>b) No	Have you heard about AED?<br>a) Yes<br>b) No	Have you ever heard AED?<br>a)Yes<br>b) No
	Adakah anda tahu bahawa melakukan AED dengan	Do you know that performing AED as soon as possible can increase chances to survive?	Do you know that by performing AED quickly can increase one's survival rate?	Do you know that performing AED as soon as possible can increase chances to survive?

	segera dapat meningkatkan peluang hidup?<br>a) Ya<br>b) Tidak	c) Yes<br>d) No	a) Yes<br>b) No	a) Yes<br>b) No
	Adakah anda tahu lokasi AED berdekatan dengan rumah atau tempat kerja anda?<br>a) Ya<br>b) Tidak	Do you know AED location which is nearby your house or nearby your work place?<br>a) Yes<br>b) No	Do you know the location of AED nearby your housing area or office?<br>a) Yes<br>b) No	Do you know AED location which is nearby your house or your work place?<br>a) Yes<br>b) No
	Dimanakah bahagian yang betul untuk meletakkan pad AED?<br>a) A-A<br>b) B-B	*What is the right position to place AED pad?<br>*Which one is the appropriate position to place AED pad?<br>a) A-A<br>b) B-B	What is the correct position to place AED pads?<br>a) A-A<br>b) B-B	What is the correct position to place AED pads?<br>a) A-A<br>b) B-B
	Adakah anda tahu terdapat arahan bersuara di dalam AED?<br>a) Ya<br>b) Tidak	Do you know that there is voice-commands/ voice instructions in AED?<br>a) Yes<br>b) No	Do you know that there are voice prompts in AED?<br>a) Yes<br>b) No	Do you know that there are voice prompts in AED?<br>a) Yes<br>b) No
**Training**	Jumlah sesi pembelajaran CPR dihadiri	Numbers of attended CPR learning sessions	Total number of attended learning CPR sessions	Total number of attended learning CPR sessions
	Jarak masa daripada sesi pembelajaran CPR terakhir	Time interval from the last CPR learning session	Time interval from the last attended CPR learning session	Time interval from the last CPR learning session
**Attitude**	Melakukan resusitasi/bantuan pernafasan dapat membantu saya menyelamatkan nyawa.	Performing respiratory assistance can help me to save life	Performing rescue breathing allows me to save one's life.	Performing respiratory assistance can help me to save life
	Jika saya meminta bantuan untuk seseorang yang mengalami serangan jantung ketika saya berada di sebuah bandar besar (dimana ambulans dapat datang	If I ask for help for a person who is experiencing heart attack when I am at an urban city (which ambulance can come quickly), chances for the patient to survive would not	If I seek assistance for someone who has cardiac arrest while I am in a big city, where ambulance could come immediately, chances for the victim to survive remain	If I ask for help for a person who is experiencing heart attack when I am at an urban city (which ambulance can come quickly), chances for the patient to survive would

	dengan segera), peluang mangsa untuk hidup tidak akan berubah jika saya melakukan bantuan pernafasan atau tidak.	change either I perform breathing/ respiratory assistance or not.	unchange, whether or not I perform rescue breathing.	not change either I perform breathing/ respiratory assistance or not.
	Dengan memberi bantuan pernafasan terhadap orang yang tidak dikenali, saya berisiko untuk mendapat penyakit (sebagai contoh AIDS atau hepatitis)	By performing respiratory assistance towards a stranger, it is risky for me to be infected by diseases (such as AIDS and Hepatitis)	By performing rescue breathing to strangers, I would be contracted to diseases (for examples, AIDS and Hepatitis)	By performing respiratory assistance towards a stranger, it is risky for me to be infected by diseases (such as AIDS and Hepatitis)
	Dengan melakukan penekanan dada kepada mangsa yang mengalami serangan jantung, saya mungkin akan lebih mencederakan dia (contohnya mematahkan tulang rusuk dengan tidak sengaja yang boleh menyebabkan kecederaan kepada paru-paru). Ini boleh membahayakan nyawa. Jadi adalah lebih baik untuk saya tidak melakukan bantuan pernafasan dan terus dapatkan pertolongan.	By performing chest compression to a victim/ patient who experiences heart attack, I may hurt him/her. (For example accidentally breaking ribs which may cause injury to lungs). This can endanger life. Therefore, it is better for me not to perform any respiratory/ breathing assistance and get help immediately..	By performing chest compression to cardiac arrest victim, I am more likely leading him to serious injury (for examples, rib fracture that can cause damage to lungs). This would endanger life. Thus, it is best for me to not performing rescue breathing and directly seek proper assistance.	By performing chest compression to heart attack victim, I am more likely leading him to serious injury (for example accidentally breaking ribs which may cause injury to lungs). This would endanger life. Thus, it is best for me to not performing respiratory/ breathing assistance and directly seek proper assistance.
	Jika seseorang tidak pasti cara yang betul untuk melakukan penekanan dada tetapi terjumpa seseorang yang tidak sedarkan diri dan tidak bernafas, adalah lebih baik untuk menunggu ambulans daripada memulakan	If a person is unsure on the right way to do chest compression and accidentally meet someone who faints and does not breath, it is better to wait for ambulance than performing respiratory assistance.He/ She can cause	If one is unsure on the proper way of performing chest compression but accidentally meet someone who is unconcious, it is better to wait for the ambulance rather than performing rescue breathing to him. One could worsen the	If a person is unsure on the right way to do chest compression and accidentally meet someone who is unconcious, does not breath, it is better to wait for ambulance than performing respiratory assistance. One

	resusitasi/bantuan pernafasan. Dia boleh mencederakan mangsa jika melakukan resusitasi/bantuan pernafasan dengan cara yang salah.	injury to the victim/patient by performing inaccurate respiratory assistance.	victim's condition if it is done poorly.	could worsen the victim's condition if it is done poorly.
	Jika saya melakukan bantuan pernafasan dan mencederakan mangsa, saya boleh dikenakan tindakan undang-undang.	If I can perform respiratory assistance and I cause injury to the victim, I am subjected to legal action.	If I perform rescue breathing and injure the victim, I can be fined.	If I can perform respiratory assistance and I cause injury to the victim, I am subjected to legal action.
	Saya akan berasa bangga apabila dapat memberi bantuan pernafasan kepada seseorang.	I feel proud when I can perform respiratory assistance to a person.	I am delighted when performing rescue breathing to someone needed.	I feel proud when I can perform respiratory assistance to a person.
	Dapat melakukan bantuan pernafasan adalah perkara yang baik untuk masyarakat.	Able to perform respiratory assistance I something good to the community.	Being able to perform rescue breathing is a great act for the community.	Able to perform respiratory assistance is something good to the community.
**Perceived Norms**	Ahli keluarga dan kawan saya akan berasa bangga jika saya melakukan bantuan pernafasan.	My family members and friends will feel proud if I perform respiratory assistance	My family members and friends would be delighted if I perform rescue breathing.	My family members and friends will feel proud if I perform respiratory assistance
	Jika ada ahli keluarga atau kawan mengalami serangan jantung, mereka akan mahukan saya untuk melakukan bantuan pernafasan untuk cuba menyelamatkan mereka.	If my family members and friends experience heart attack, they will want me to do respiratory assistance to save them.	If my family members or friends experience cardiac arrest, they would want me to perform rescue breathing to save their lives.	If my family members and friends experience cardiac arrest, they will want me to do respiratory assistance to save their lives.
	Jika suatu hari saya menyaksikan serangan jantung, mangsa tersebut berkemungkinan besar (secara statistik) adalah dalam	If one day, I witness a heart attack, the victim is most probably (statistically) are among close relatives (family members or friends)	If one day, I witness cardiac arrest, the victim probably could be (statically) from my close relatives (family members and friends)	If one day, I witness a cardiac arrest, the victim is most probably (statistically) are among close relatives (family members or friends)

	kalangan saudara terdekat (ahli keluarga atau rakan).			
	Jika saya berada di dalam stesen kereta api yang sesak dan seseorang jatuh pengsan dihadapan saya, adalah lebih baik saya tunggu dan lihat jika seseorang yang lebih terlatih dalam memberi resusistasi/bantuan pernafasan sebelum saya melakukan tindakan.	If I am at a train station and someone faints in front of me, it's better for me to wait and see if there is someone who is better trained in respiratory assistance before I take any action.	If I im in a crowded train station and someone loses conciousness in front of me, it is better for me to wait and see if there is any trained personnel in performing rescue breathing before I conduct it myself.	If I Im in a crowded train station and someone loses conciousness in front of me, it is better for me to wait and see if there is any trained personnel in performing respiratory assistance before I take any action.
**Self -Efficacy**	Sedia tahu nombor panggilan ambulan di Malaysia. Dengan itu, saya dapat melakukan tindakan segera jika saya berhadapan dengan seseorang yang tidak sedarkan diri dan tidak bernafas.	Have already known contact number of ambulance in Malaysia. Therefore, I can perform quick action if I encounter someone who is unconscious and does not breath	I am aware of emergency contact number for ambulance in Malaysia. Hence, I am able to make quick action if I experience situation where one loses his conciousness and unable to breathe.	Have already known contact number of ambulance in Malaysia. Therefore, I can perform quick action if I encounter someone who is unconscious and does not breath
	Saya rasa saya boleh melakukan resusitasi/bantuan pernafasan.	I think I am able to do respiratory assistance.	I am able to perform rescue breathing.	I think I am able to do respiratory assistance.
	Saya boleh mengenalpasti jika seseorang memerlukan resusitasi/bantuan pernafasan.	I can identify if anyone needs respiratory assistance	I can identify if one needs rescue breathing.	I can identify if anyone needs respiratory assistance
	Hanya professional kesihatan yang boleh melakukan resusitasi/bantuan pernafasan yang berkesan.	Only health professional/ medical experts can perform respiratory assistance.	Only healthcare personnel can perform rescue breathing effectively	Only healthcare personnel can perform respiratory assistance effectively
	Saya tahu bagaimana cara untuk melakukan bantuan pernafasan. Dengan itu saya boleh membantu mangsa serangan jantung dengan efisyen.	I know how to perform respiratory assistance. Because of that I can help heart attack victim/ patient efficiently.	I know ways to perform rescue breathing. Hence, I can help cardiac arrest victim efficiently.	I know ways to perform respiratory assistance. Hence, I can help cardiac arrest victim efficiently.

**Intention**	Dalam situasi kecemasan, adakah anda akan melakukan CPR kepada orang yang tidak dikenali?	In emergency situation, will you perform CPR to a stranger?	In emergency situation, will you perform CPR to strangers?	In emergency situation, will you perform CPR to a stranger?
	Dalam situasi kecemasan, adakah anda akan melakukan CPR kepada mangsa trauma?	In emergency situation, will you perform traumatized victim?	In emergency situation, will you perform CPR to traumatised victims?	In emergency situation, will you perform CPR to a traumatized victim?
	Dalam situasi kecemasan, adakah anda akan melakukan CPR kepada kanak-kanak?	In emergency situation, will you perform CPR to kids?	In emergency situation, will you perform CPR to children?	In emergency situation, will you perform CPR to children?
	Dalam situasi kecemasan, adakah anda akan melakukan CPR kepada orang tua?	In emergency situation, will you perform CPR to elderly?	In emergency situation, will you perform CPR to the elderly?	In emergency situation, will you perform CPR to the elderly?
	Dalam situasi kecemasan, adakah anda akan melakukan CPR kepada saudara atau ahli keluarga?	In emergency situation, will you perform CPR to relatives and family members?	In emergency situation, will you perform CPR to your relatives or family members?	In emergency situation, will you perform CPR to relatives and family members?
	Dalam situasi kecemasan, adakah anda akan menggunakan AED?	In emergency situation, will you be using AED?	In emergency situation, will you use AED?	In emergency situation, will you be using AED?

*The red highlight colour represents the discrepancies and issues addressed between translators

*The green highlight colour represents the synthesis of intensive discussions among the experts and translators

Supplementary Material Table S2. Summarizes the information of the content validity expert review panel of this instrument

No	Initial	Expertise	Gender	Years of Experience	Position and Place of Work
1.	Dr. A	Transthoracic and Transesophageal (TEE), Echocardiography, Coronary Angiogram & Angioplasty, Assessment of Valvular Heart Disease, Assessment of Adult Congenital Heart Disease, Interventional Echocardiography in Left Ventricular Assist Device (LVAD), Mitraclip, Percutaneous Transvenous Mitral Commissurotomy (PTMC), Adult Cardiology and Adult Cardiology for Heart Disease in Pregnancy	Male	16 years	Cardiologist, National Heart Institute
2.	Dr B	Emergency Critical Care and Critical Ultrasound	Male	24 years	Emergency Consultant and Head of Melaka State Emergency Medical Services
3.	Dr C	Doctor of Philosophy in Curriculum Development - Paramedics	Male	28 years	Senior Lecturer, Department of Emergency Medicine, Universiti Kebangsaan Malaysia
4.	Mr. D	Diploma in Medical Assistant, Post basic in Advanced Emergency Medical and Trauma Care (AEMTC)	Male	16 years	Instructor and Member of the resuscitation committee of Melaka Hospital
5.	Mrs. E	Degree in Health Education	Female	23 years	Head of Patient Education Unit, Melaka Hospital

Supplementary Material Table S3. Conceptual and operational definitions of FIXED

Construct<br>(Conceptual Definition)	Aspect<br>(Operational Definition)
**Knowledge**<br>Basic knowledge of CPR and AED and community understanding of the practical application of CPR and AED to improve the survival of patients with cardiac arrest	- Knowing CPR can be performed outside a hospital setting<br>- Knowing CPR to be performed without mouth-to-mouth resuscitation<br>- Perform CPR without certification<br>- Correct sequence to perform CPR<br>- Conditions for which CPR must be performed<br>- The right location to perform chest compression<br>- Chest compression rate<br>- Depth of compression that should be performed during chest compression<br>- Ever heard of AED<br>- Know that prompt use of an AED can increase survival<br>- Know the location of an AED nearest to home or workplace<br>- Correct position for placement of the AED pads<br>- Know there is voice prompt in the AED
**Training**<br>Received training related to increased confidence and willingness to perform CPR and use an AED effectively	- Number of CPR learning sessions that have been attended<br>- Last CPR learning session
**Perception**<br>An individual's subjective experience and awareness determines how a person participates in a particular behaviour	**Implementation Strategies of the AED**<br>- The AED is clearly visible<br>- The signage that shows the location of the AED is clear<br>- The AED is located in a location that is easily accessible at all times (including after office hours)<br>- The steps in the AED instructional poster on how to use the AED are easy to follow<br>- The AED is located at a secure site<br>**Community's perception on the importance of CPR and AED**<br>- CPR & AED are important in saving life<br>- It is important for an AED to be available in the place where I work.<br>- Using an AED is important on any unresponsive victims<br>- Person who handles an AED requires formal training.<br>- AED practice drills should be performed on a regular basis

**Attitude**<br>It is the attitude of an individual that can influence their willingness based their own assessment positively or negatively	- Thinking that performing resuscitation could save a life<br>- Knowing the importance of starting a resuscitation before EMS arrival<br>- Not being afraid of disease transmission<br>- Not being afraid of hurting the victim by performing CPR<br>- Not being afraid of worsening the victim's condition<br>- Not being afraid of legal action<br>- Being proud of performing resuscitation successfully<br>- Belief that knowing CPR is important for the society
**Perceived Norms**<br>Social stress received from people around that influences an individual's willingness behaviour whether to perform CPR and use an AED or vice versa	- Belief that relatives would be proud if the participant performed resuscitation<br>- Belief that relatives want the subject to resuscitate them if needed<br>- Knowing that relatives are the most likely victim<br>- Diffusion of responsibility
**Self -Efficacy**<br>A person's confidence in their ability to perform a particular behaviour by considering the possibility that there are factors that influence or limit it	- Knowledge of the emergency number Feeling able to resuscitate<br>- Feeling able to recognize a cardiac arrest<br>- Not believing that only health care professionals can adequately perform resuscitation<br>- Knowing how to perform a resuscitation
**Intention**<br>A person's willingness to perform a behaviour that is to perform CPR and use an AED when an actual cardiac arrest occurs	- Perform CPR on a stranger<br>- Perform CPR on a victim of trauma<br>- Perform CPR on a child<br>- Perform CPR on an elderly person<br>- Perform CPR on a relative or family member<br>- Using an AED
**Behaviour of Willingness to Perform CPR and Use an AED**<br>The confidence of a person to perform CPR and use an AED that is to immediately perform CPR and use an AED can save lives	- Confident to perform CPR<br>- Confident to use an AED<br>- Confident in recognizing victim with no signs of life<br>- Confident to use an AED on an unresponsive victim
**Barriers That Affect Behaviour from Performing CPR and Using an AED**<br>Barriers that need to be identified and addressed to enable the community to take action during emergencies. This allows the community to overcome these barriers and actively decide to take action to perform CPR and use an AED	**Concerns of injuring victims during CPR and AED**<br>- Concerned in getting infection from the victim when performing CPR<br>- Concerned in injuring the victim when performing CPR<br>- Concerned in injuring myself when performing CPR

	- Concerned in injuring the victim if I use an AED device during CPR<br>- Concerned in injuring myself if I use an AED device during CPR<br>**Concerns of legality in performing CPR and AED**<br>- Concerned that I might be sued if I perform emergency CPR inappropriately<br>- Concerned that I might be sued if I used an AED inappropriately

Supplementary Material Table S4. The relevance ratings on the item scale by five experts

Item	Expert 1	Expert 2	Expert 3	Expert 4	Expert 5	Expert in Agreement	I-CVI	Interpretation
1	1.00	1.00	1.00	1.00	1.00	5	1.00	Appropriate
2	1.00	1.00	1.00	1.00	1.00	5	1.00	Appropriate
3	1.00	1.00	1.00	1.00	1.00	5	1.00	Appropriate
4	1.00	1.00	1.00	1.00	1.00	5	1.00	Appropriate
5	1.00	1.00	1.00	1.00	1.00	5	1.00	Appropriate
6	1.00	1.00	1.00	1.00	1.00	5	1.00	Appropriate
7	1.00	1.00	1.00	1.00	1.00	5	1.00	Appropriate
8	1.00	1.00	1.00	1.00	1.00	5	1.00	Appropriate
9	1.00	1.00	1.00	1.00	1.00	5	1.00	Appropriate
10	1.00	1.00	1.00	1.00	1.00	5	1.00	Appropriate
11	1.00	1.00	1.00	1.00	1.00	5	1.00	Appropriate
12	1.00	1.00	1.00	1.00	1.00	5	1.00	Appropriate
13	1.00	1.00	1.00	1.00	1.00	5	1.00	Appropriate
14	1.00	1.00	1.00	1.00	1.00	5	1.00	Appropriate
15	1.00	1.00	1.00	1.00	1.00	5	1.00	Appropriate
16	1.00	1.00	1.00	1.00	1.00	5	1.00	Appropriate
17	1.00	1.00	1.00	1.00	1.00	5	1.00	Appropriate
18	1.00	1.00	1.00	1.00	1.00	5	1.00	Appropriate
19	1.00	1.00	1.00	1.00	1.00	5	1.00	Appropriate
20	1.00	1.00	1.00	1.00	1.00	5	1.00	Appropriate
21	1.00	1.00	1.00	1.00	1.00	5	1.00	Appropriate
22	1.00	1.00	1.00	1.00	1.00	5	1.00	Appropriate
23	1.00	1.00	1.00	1.00	1.00	5	1.00	Appropriate
24	1.00	1.00	1.00	1.00	1.00	5	1.00	Appropriate
25	1.00	1.00	1.00	1.00	1.00	5	1.00	Appropriate
26	1.00	1.00	1.00	1.00	1.00	5	1.00	Appropriate
27	1.00	1.00	1.00	1.00	0.00	4	0.80	Appropriate
28	1.00	1.00	1.00	1.00	0.00	4	0.80	Appropriate
29	1.00	1.00	1.00	1.00	0.00	4	0.80	Appropriate
30	1.00	1.00	1.00	1.00	1.00	5	1.00	Appropriate
31	1.00	1.00	1.00	1.00	0.00	4	0.80	Appropriate
32	1.00	1.00	1.00	1.00	0.00	4	0.80	Appropriate
33	1.00	1.00	1.00	1.00	1.00	5	1.00	Appropriate
34	1.00	1.00	1.00	1.00	0.00	4	0.80	Appropriate
35	1.00	1.00	1.00	1.00	1.00	5	1.00	Appropriate
36	1.00	1.00	1.00	1.00	1.00	5	1.00	Appropriate
37	1.00	1.00	1.00	1.00	1.00	5	1.00	Appropriate
38	1.00	1.00	1.00	1.00	1.00	5	1.00	Appropriate
39	1.00	1.00	1.00	1.00	1.00	5	1.00	Appropriate
40	1.00	1.00	1.00	1.00	1.00	5	1.00	Appropriate
41	1.00	1.00	1.00	1.00	1.00	5	1.00	Appropriate
42	1.00	1.00	1.00	1.00	1.00	5	1.00	Appropriate
43	1.00	1.00	1.00	1.00	1.00	5	1.00	Appropriate
44	1.00	1.00	1.00	1.00	1.00	5	1.00	Appropriate
45	1.00	1.00	1.00	1.00	1.00	5	1.00	Appropriate
46	1.00	1.00	1.00	1.00	1.00	5	1.00	Appropriate

47	1.00	1.00	1.00	1.00	1.00	5	1.00	Appropriate
48	1.00	1.00	1.00	1.00	1.00	5	1.00	Appropriate
49	1.00	1.00	1.00	1.00	1.00	5	1.00	Appropriate
50	1.00	1.00	1.00	1.00	1.00	5	1.00	Appropriate
51	1.00	1.00	1.00	1.00	1.00	5	1.00	Appropriate
52	1.00	1.00	1.00	1.00	1.00	5	1.00	Appropriate
53	1.00	1.00	1.00	1.00	1.00	5	1.00	Appropriate
54	1.00	1.00	1.00	1.00	1.00	5	1.00	Appropriate
55	1.00	1.00	1.00	1.00	1.00	5	1.00	Appropriate
56	1.00	1.00	1.00	1.00	1.00	5	1.00	Appropriate
57	1.00	1.00	1.00	1.00	1.00	5	1.00	Appropriate
58	1.00	1.00	1.00	1.00	1.00	5	1.00	Appropriate
59	1.00	1.00	1.00	1.00	1.00	5	1.00	Appropriate
**Average proportion of items judged as relevance across five experts**							**0.98**	
**Proportion relevance**	**1.00**	**1.00**	**1.00**	**1.00**	**0.90**			

Note:
Interpretation of I-CVI: If the I-CVI is higher than 79 percent, the item will be appropriate. If it’s between 70 and 79 percent, it needs revision. If less than 70 percent, it should be eliminated.

Supplementary Material Table S5. The Fleiss Kappa Index based on a suitability level assessment by five experts

n	1	2	3	4	Total	P_i
1	0	0	0	5	5	1.00
2	0	0	2	3	5	0.40
3	0	0	1	4	5	0.60
4	0	0	0	5	5	1.00
5	0	0	0	5	5	1.00
6	0	0	0	5	5	1.00
7	0	0	1	4	5	0.60
8	0	0	1	4	5	0.60
9	0	0	0	5	5	1.00
10	0	0	0	5	5	1.00
11	0	0	0	5	5	1.00
12	0	0	0	5	5	1.00
13	0	0	0	5	5	1.00
14	0	0	0	5	5	1.00
15	0	0	0	5	5	1.00
16	0	0	1	4	5	0.60
17	0	0	0	5	5	1.00
18	0	0	0	5	5	1.00
19	0	0	2	3	5	0.40
20	0	0	0	5	5	1.00
21	0	0	0	5	5	1.00
22	0	0	0	5	5	1.00
23	0	0	0	5	5	1.00
24	0	0	1	4	5	0.60
25	0	0	1	4	5	0.60
26	0	0	0	5	5	1.00
27	1	0	2	2	4	0.20
28	1	0	1	3	4	0.30
29	1	0	3	1	4	0.30
30	0	0	3	2	5	0.40
31	1	0	0	4	4	0.60
32	1	0	0	4	4	0.60
33	0	0	0	5	5	1.00
34	1	0	0	4	4	0.60
35	0	0	0	5	5	1.00
36	0	0	0	5	5	1.00
37	0	0	0	5	5	1.00
38	0	0	1	4	5	0.60
39	0	0	0	5	5	1.00
40	0	0	0	5	5	1.00
41	0	0	0	5	5	1.00
42	0	0	1	4	5	0.60
43	0	0	0	5	5	1.00
44	0	0	0	5	5	1.00
45	0	0	0	5	5	1.00
46	0	0	0	5	5	1.00
47	0	0	0	5	5	1.00
48	0	0	0	5	5	1.00

49	0	0	0	5	5	1.00
50	0	0	0	5	5	1.00
51	0	0	0	5	5	1.00
52	0	0	0	5	5	1.00
53	0	0	0	5	5	1.00
54	0	0	0	5	5	1.00
55	0	0	0	5	5	1.00
56	0	0	0	5	5	1.00
57	0	0	0	5	5	1.00
58	0	0	0	5	5	1.00
59	0	0	0	5	5	1.00
**Total**	**6**	**0**	**21**	**268**	**295**	**50.60**
P_j	**0.020**	**<0.001**	**0.071**	**0.908**		

Based on Supplementary Material 4, number of items N = 59, number of experts n = 5, total of all cells pj = N x n = 59 x 5 = 295. The formula below shows Fleiss Kappa calculations for this instrument.

Total P_i $\quad \sum_{i=1}^{N} P_i$ $\quad$ = 1.00 + 0.40 + 0.60 + 1.00 + ... + 1.00 + 1.00 + 1.00 = 50.60

Total P_i = 50.60

$$\bar{P} = \frac{1}{N}\sum_{i=1}^{N} P_i \quad = \frac{1}{59}(50.60) = 0.858$$

Total $\bar{P}$ = 0. 858

$$\bar{P}_e = \sum_{j=1}^{k} p_j^2 \quad = 6^2 + 0^2 + 21^2 + 268^2 = 0.831$$

Total $\bar{P}e$ = 0.831

$$\kappa = \frac{\bar{P} - \bar{P}_e}{1 - \bar{P}_e} \quad = \frac{0.858 - 0.831}{1 - 0.831} = 0.159$$

Kappa Value = 0.159

Supplementary Material Table S6: Communalities values for all items

1.	The AED is clearly visible	1.000	0.672
2.	The signage that shows the location of the AED is clear	1.000	0.822
3.	The AED is located in a location that is easily accessible at all times (including after office hours)	1.000	0.778
4.	The steps in the AED instructional poster on how to use the AED are easy to follow	1.000	0.760
5.	The AED is located at a secure site	1.000	0.769
6.	CPR & AED are important in saving life	1.000	0.693
7.	It is important for an AED to be available in the place where I work.	1.000	0.680
8.	Using an AED is important on any unresponsive victims	1.000	0.500
9.	Person who handles an AED requires formal training.	1.000	0.512
10.	AED practice drills should be performed on a regular basis	1.000	0.487
11.	Thinking that performing resuscitation could save a life	1.000	0.598
12.	Knowing the importance of starting a resuscitation before EMS arrival	1.000	0.296
13.	Not being afraid of disease transmission	1.000	0.498
14.	Not being afraid of hurting the victim by performing CPR	1.000	0.540
15.	Not being afraid of worsening the victim's condition	1.000	0.597
16.	Not being afraid of legal action	1.000	0.554
17.	Being proud of performing resuscitation successfully	1.000	0.689
18.	Belief that knowing CPR is important for the society	1.000	0.662
19.	Belief that relatives would be proud if the participant performed resuscitation	1.000	0.679
20.	Belief that relatives want the subject to resuscitate them if needed	1.000	0.630
21.	Knowing that relatives are the most likely victim	1.000	0.555
22.	Diffusion of responsibility	1.000	0.454
23.	Knowledge of the emergency number	1.000	0.524
24.	Feeling able to resuscitate	1.000	0.735
25.	Feeling able to recognize a cardiac arrest	1.000	0.748
26.	Not believing that only health care professionals can adequately perform resuscitation	1.000	0.533
27.	Knowing how to perform a resuscitation	1.000	0.758
28.	Perform CPR on a stranger	1.000	0.739
29.	Perform CPR on a victim of trauma	1.000	0.464
30.	Perform CPR on a child	1.000	0.720
31.	Perform CPR on an elderly person	1.000	0.783
32.	Perform CPR on a relative or family member	1.000	0.763
33.	Using an AED	1.000	0.582
34.	Confident to perform CPR	1.000	0.851
35.	Confident to use an AED	1.000	0.853
36.	Confident in recognizing victim with no signs of life	1.000	0.733
37.	Confident to use an AED on an unresponsive victim	1.000	0.842
38.	Concerned in getting infection from the victim when performing CPR	1.000	0.613

39.	Concerned in injuring the victim when performing CPR	1.000	0.702
40.	Concerned in injuring myself when performing CPR	1.000	0.701
41.	Concerned in injuring the victim if I use an AED device during CPR	1.000	0.733
42.	Concerned in injuring myself if I use an AED device during CPR	1.000	0.626
43.	Concerned that I might be sued if I perform emergency CPR inappropriately	1.000	0.772
44.	Concerned that I might be sued if I used an AED inappropriately	1.000	0.723
